# Monocyte biomarkers define sargramostim treatment outcomes for Parkinson's disease

**DOI:** 10.1002/ctm2.958

**Published:** 2022-07-08

**Authors:** Mai M. Abdelmoaty, Jatin Machhi, Pravin Yeapuri, Farah Shahjin, Vikas Kumar, Katherine E. Olson, R. Lee Mosley, Howard E. Gendelman

**Affiliations:** ^1^ Department of Pharmaceutical Sciences College of Pharmacy University of Nebraska Medical Center Omaha Nebraska USA; ^2^ Therapeutic Chemistry Department Pharmaceutical and Drug Industries Research Institute National Research Centre Giza Egypt; ^3^ Department of Pharmacology and Experimental Neuroscience College of Medicine University of Nebraska Medical Center Omaha Nebraska USA; ^4^ Mass Spectrometry and Proteomics Core University of Nebraska Medical Center Omaha Nebraska USA

**Keywords:** Parkinson's disease, GM‐CSF, monocytes, scRNA‐seq, proteomics, biomarkers

## Abstract

**Background:**

Dysregulation of innate and adaptive immunity heralds both the development and progression of Parkinson's disease (PD). Deficits in innate immunity in PD are defined by impairments in monocyte activation, function, and pro‐inflammatory secretory factors. Each influences disease pathobiology.

**Methods and Results:**

To define monocyte biomarkers associated with immune transformative therapy for PD, changes in gene and protein expression were evaluated before and during treatment with recombinant human granulocyte‐macrophage colony‐stimulating factor (GM‐CSF, sargramostim, Leukine^®^). Monocytes were recovered after leukapheresis and isolation by centrifugal elutriation, before and 2 and 6 months after initiation of treatment. Transcriptome and proteome biomarkers were scored against clinical motor functions. Pathway enrichments from single cell‐RNA sequencing and proteomic analyses from sargramostim‐treated PD patients demonstrate a neuroprotective signature, including, but not limited to, antioxidant, anti‐inflammatory, and autophagy genes and proteins (LRRK2, HMOX1, TLR2, TLR8, RELA, ATG7, and GABARAPL2).

**Conclusions:**

This monocyte profile provides an “early” and unique biomarker strategy to track clinical immune‐based interventions, but requiring validation in larger case studies.

## Graphical abstract

Graphical highlights: Clinical and pathobiological diversity of Parkinson's disease (PD) limits therapeutic disease monitoring. Transformation of innate immunity can affect PD progression. Granulocyte‐macrophage colony‐stimulating factor (GM‐CSF, sargramostim, Leukine^®^) transformation of monocyte‐macrophage immunity can halt PD motor signs. Transcriptomic and proteomic monocyte biomarkers from sargramostim‐treated PD patients may guide effective therapy.

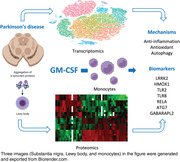

## 1 BACKGROUND

Parkinson's disease (PD) is a progressive neurodegenerative disorder characterized by the gradual loss of dopaminergic neurons from the substantia nigra pars compacta (SNpc). The dominant presence of *α*‐synuclein (*α*‐syn) aggregates in intracellular inclusions within the central nervous system (CNS) and in peripheral tissues is a principal disease driver, while providing a signature for systemic multi‐organ disease.^1^, ^2^ Linkages between PD and innate (monocyte and microglia)^3^ and adaptive (T cell) immunity, and inflammation are well‐established.^4^ The PD pathogenesis involves impaired protein clearance, mitochondrial dysfunction, and inflammation.^5^ All affect dopaminergic neuronal function with secondary effects on noradrenergic, glutamatergic, serotonergic, and adenosine neuronal vitality.^6^ Disease is highlighted by release of *α*‐syn aggregates into systemic circulation and consequent monocyte activation and brain entry,^7^ and deficits of neuroimmune homeostasis,^8^ Based on these findings, efforts to restore brain homeostasis are attractive targets for PD‐modifying therapies.

In PD, regulatory T cells (Treg) are decreased in number and display impaired suppression of T cell proliferation.^9^ One PD therapeutic strategy focuses on immune transformation. Notably, balanced transformation of M1 microglia/macrophages/monocytes to M2/M0 phenotypes and effector T cells (Teff) to Treg, and interactions between the two lineages are of putative clinical benefit for PD as well as for Alzheimer's disease (AD), traumatic brain injury (TBI), stroke, and amyotrophic lateral sclerosis (ALS).^10^, ^11^ Restoration of Treg numbers and function by immune modulatory agents leads to control of neuroinflammation and neuroprotection.^12^ This was demonstrated for granulocyte‐macrophage colony‐stimulating factor (GM‐CSF, sargramostim, Leukine^®^),^13^, ^14^, ^15^, ^16^ vasoactive intestinal peptide (VIP),^17^ and by *ex vivo* immune transformation.^18^ Moreover, pre‐clinical and clinical studies of immune modulatory therapies have shown benefits for PD, AD, ALS, and stroke as well as multiple sclerosis (MS) and rheumatoid arthritis (RA),^13^, ^14^, ^15^, ^16^, ^19^, ^20^, ^21^ and raises the possibility that neuroprotective therapies can supplement symptomatic disease control.

To evaluate innate responses in immune modulatory therapy in PD, we evaluated the transcriptional and translational changes of monocytes during sargramostim treatment of PD subjects.^14^ The studies seek a cogent explanation for linkages between innate immune activation, inflammation, and adaptive Teff to Treg transformation for disease. We posit that uncovering such monocyte‐linked neuroprotective mechanisms provides predictive and/or monitoring biomarkers for therapeutic responses and possibly disease progression.^22^ With these goals in mind, blood monocytes were isolated from 5 PD patients by leukapheresis and centrifugal elutriation, before and after initiation of sargramostim therapy. The recovered cells were subjected to a battery of transcriptomic and proteomic analyses yielding therapeutic signatures of dysregulated anti‐inflammatory, antioxidant, and autophagy genes and proteins. We conclude that the nature of monocyte functions in PD are both associated with disease pathobiology and can be harnessed to affect disease progression and clinical therapeutic responses.

## 2 METHODS AND MATERIALS

### 2.1 Study design and population

The study was an unblinded, open‐label, single‐centre phase 1 clinical trial performed at the University of Nebraska Medical Center (UNMC), Omaha, NE, USA.^14^ All patients were recruited from the Omaha metropolitan area and treated for 12 months between January, 2019 and July, 2020. The study was designed to test safety, tolerability, and biomarker discovery of sargramostim regimen of 3 µg/kg/day (5 days on and 2 days off).^14^ Eligibility criteria were 35 to 85 years of age with PD signs and symptoms that included asymmetric bradykinesia, resting tremor, and/or muscle rigidity persisting for longer than 3 years with less than stage 4 on the Hoehn and Yahr disease scale, indicating an intermediate stage of the disease.^14^ Exclusion criteria included inability to undergo leukapheresis, poor venous access, use of a wheelchair, walker, and/or cane, corticobasal degeneration, multiple system atrophy, unilateral Parkinsonism of >3 years, prior head injury, stroke, brain surgery including deep brain stimulation, a family history of >1 blood relative with PD, mental illness, cognitive impairment, autoimmune, systemic inflammatory or hematologic diseases, past treatment with sargramostim, current treatment with neuroleptics or lithium, past immunosuppressive treatments, and known allergies to colony‐stimulating factors (CSFs) or yeast‐derived products.^14^ PD patients underwent three pre‐treatment monthly interval appointments to determine baseline immune, hematologic, and metabolic profiles.^14^ On the third visit, subjects self‐administered sargramostim at 3 µg/kg/day (5 days on and 2 days off) subcutaneously for 12 months; returning for clinical assessments every 4 weeks. The primary neurologist performed all Unified Parkinson's Disease Rating Scale (UPDRS) assessments during each appointment.^14^ Before treatment initiation and at 2 and 6 months after initiation, subjects underwent leukapheresis. The procedure was completed to enrich peripheral blood mononuclear cells in order to obtain sufficient numbers of monocytes for proteomic analysis and scRNA‐seq test analyses.

### 2.2 Monocyte isolation

Blood samples were collected from the subjects before starting the treatment as well as after 2 and 6 months of treatment. Whole blood was collected into tubes containing ethylenediaminetetraacetic acid (EDTA) and monocytes were isolated by centrifugal elutriation following established protocol in our laboratories (Materials and Methods in the Supplementary information file). Isolated monocytes were stored in freezing medium [foetal bovine serum (FBS) + 10% dimethyl sulfoxide (DMSO)] and kept in liquid nitrogen until processing for proteomic and transcriptomic analyses. The monocyte samples were processed for different assays within 6 months after storage in liquid nitrogen. After thawing the samples, recovery was 90%–95% of the number of cryopreserved cells (10 × 10^6^ cells/vial) and microscopic examination showed normal cellular morphology. The phenotype of monocytes was confirmed by measures of monocyte cell surface markers CD14 and CD16 by scRNA‐seq.

### 2.3 Quantitative proteomics by label‐free quantification

Frozen monocyte samples were thawed on ice, centrifuged to remove the freezing medium, washed with phosphate buffered saline (PBS), then lysed on ice with 250 µl of 2% w/v sodium dodecyl sulphate (SDS) (Fisher Scientific, BP166‐100) in 100 mM Tris‐HCl (Millipore Sigma, 10812846001) and 100 mM dithiothreitol (Millipore Sigma, 10197777001), pH 7.6, supplemented with a protease and phosphatase inhibitor cocktail (Millipore Sigma, PPC1010). Lysate was collected in 1.7 ml microcentrifuge tubes and vortexed briefly to disperse the cells. One microliter of Benzonase Nuclease (Millipore Sigma, E1014) was added to each sample to breakdown DNA, then samples were vortexed on maximum speed for >12 min. Samples were boiled at 95°C for 5 min to denature proteins. Protein concentration was determined using Pierce 660 Protein Assay kit (Thermo Fisher Scientific, 22662) with ionic detergent compatibility reagent (Thermo Fisher Scientific, 22660) following the manufacturer's instructions. Samples were processed as previously described^23^ by filter‐aided sample preparation ((FASP, Pall Life Sciences, OD010C34)) to digest 50 µg per sample. Following overnight digestion, samples were cleaned using the Oasis MCX column (Waters, 186000252) and C18 Zip‐Tips (Sigma‐Aldrich, ZTC18M960). Cleaned peptides were quantitated using NanoDrop2000 at A205. Following resuspension in 0.1% formic acid (FA), 2 µg of sample was used for label‐free quantification (LFQ) as previously described.^24^ Briefly, 2 µg of each sample was loaded onto trap column Acclaim PepMap 100 75 µm × 2 cm C18 LC Columns (Thermo Fisher Scientific) at a flow rate of 4 µl min^−1^ then separated with a Thermo RSLC Ultimate 3000 (Thermo Fisher Scientific) on a Thermo Easy‐Spray PepMap RSLC C18 75 µm × 50 cm C‐18 2 µm column (Thermo Fisher Scientific) with a step gradient of 4%–25% solvent B (0.1% FA in 80% acetonitrile) from 10–100 min and 25%–45% solvent B for 100–130 min at 300 nl min^−1^ and 50°C with a 155 min total run time. Eluted peptides were analysed by a Thermo Orbitrap Fusion Lumos Tribrid (Thermo Fisher Scientific) mass spectrometer in a data‐dependent acquisition mode. A survey full scan MS (from m/z 350–1800) was acquired in the Orbitrap with a resolution of 120 000. The automatic gain control (AGC) target for precursor ion scan (MS1) was set as 4 × 10^5^ and ion filling time set at 100 ms. The most intense ions with charge state 2–6 were isolated in 3 s cycles and fragmented using higher energy collisional dissociation fragmentation with 35% normalized collision energy and detected at a mass resolution of 30 000 at 200 m/z. The AGC target for MS/MS was set at 5 × 10^4^ and ion filling time set 60 ms dynamic exclusion was set for 30 s with a 10 ppm mass window. Protein identification was performed by searching MS/MS data against the SwissProt *Homo sapiens* protein database downloaded in March 2021 using the in‐house PEAKS X + DB search engine. The search was set up for full tryptic peptides with a maximum of two missed cleavage sites. Acetylation of protein N‐terminus and oxidized methionine were included as variable modifications and carbamidomethylation of cysteine was set as fixed modification. The precursor mass tolerance threshold was set 10 ppm and maximum fragment mass error was 0.02 Da. The significance threshold of the ion score was calculated based on a false discovery rate (FDR) of ≤1%. Quantitative data analysis was performed using Progenesis QI proteomics 4.2 (Nonlinear Dynamics). Statistical analysis was performed using ANOVA and the Benjamini–Hochberg (BH) method^25^ was used to adjust *p* values for multiple‐testing FDR.

### 2.4 Differential proteomic analyses

Proteins identified by mass spectrometry were quantified to identify differentially expressed proteins between each treatment (2‐ and 6‐months post‐treatment) and baseline (pre‐treatment) for each patient alone as well as between each treatment and control condition for all patients together. ANOVA *P* value (computed from the proteomics core) and absolute fold changes were used to identify differentially expressed proteins in different comparisons. A protein was considered to be differentially expressed if the *P* value was ≤ 0.05 and the absolute fold change was ≥2. The IPA (Qiagen) was used to identify the pathways and networks affected in 2 and 6 months following the treatment compared to baseline (before starting the treatment). Functional and pathway enrichment analyses of differentially expressed proteins were conducted using Cytoscape in conjunction with the plug‐in ClueGO^26^ to identify the enriched immune responses, biological processes, molecular functions, cellular components, Kyoto Encyclopedia of Genes and Genomes (KEGG) pathways, and Reactome pathways. The search tool for the retrieval of interacting genes/proteins (STRING) local network cluster enrichment was conducted using Cytoscape in conjunction with the plug‐in STRING Enrichment which provides critical assessments and integration of PPIs. The cut‐off used for Venn diagrams and general differential expression analysis summary was: *P* value of 0.05 and absolute fold change of 1.5. The IPA and ClueGO analyses were also performed on proteins with the same cut‐off *p* value of 0.05. For Volcano plots, the cut‐off used to add gene names to differentially expressed proteins was: absolute log_2_‐fold change *>*2 and ANOVA *P* value ≤ 0.05.

### 2.5 Single‐cell RNA‐sequencing

Cryopreserved monocyte samples (for 3 study participants having IDs 2003, 2004, and 2005) at baseline and 6 months were assessed. Cells were thawed on ice, centrifuged, and washed with PBS, then processed for single‐cell RNA‐sequencing (scRNA‐seq); as previously described,^27^ in FACS buffer (2% FBS + 0.1% NaN3 in PBS). All reagents purchased from Sigma unless otherwise specified. Single cell suspensions were quantified and viability tested using a LUNA‐FL^TM^ Dual Fluorescence Cell Counter (Logos Biosystems). Single cells were then isolated from cell suspensions (100–2,000 cells/µl) using a 10x Chromium Controller per manufacturer's suggested protocol (10x Genomics). Following cell capture, the gel beads in emulsion (GEM)/sample solution were recovered and placed into strip tubes. Reverse transcription was performed on a C1000 Touch™ Thermal Cycler (Bio‐Rad) per recommended protocol followed by cDNA amplification. Amplified products were solid phase reversible immobilization (SPRI) bead‐purified and evaluated by Fragment Analyzer (Agilent). Twenty‐five percent of the cDNA volume was subjected to fragmentation and double‐sided SPRIselect (Beckman Coulter) was used for PCR purification and clean‐up. After adaptor ligation, SPRI clean‐up was performed and PCR amplification using sample specific indexes for each sample was completed. PCR products were purified, quantified, and library size distribution determined by Fragment Analyzer. Libraries were sequenced per the manufacturer's suggested parameters on a NextSeq500 sequencer to an average depth of 50,000 reads per cell.

### 2.6 Droplet digital polymerase chain reaction assay

Frozen monocyte samples were thawed on ice, centrifuged to remove the freezing medium, and washed with PBS, then total RNA was isolated using RNeasy Mini Kit (Qiagen, 74104), and cDNA was generated utilizing RevertAid First Strand cDNA synthesis kit (Thermo Fisher Scientific, K1622). Copy numbers of genes of interest normalized to those of a housekeeping gene (HPRT1)/µl reaction were determined by droplet digital polymerase chain reaction (ddPCR) (QX200 Droplet Digital PCR System, Bio‐Rad) from 10 ng of template cDNA using ddPCR Supermix for Probes (No dUTP) (Bio‐Rad, 1863024), ddPCR Copy Number Variation Assays (FAM) (Bio‐Rad, 10042958) for leucine rich repeat kinase 2 (LRRK2) (dHsaCNS305132784), heme oxygenase 1 (HMOX1) (dHsaCNS341530792), toll‐like receptor 2 (TLR2) (dHsaCNS915093309), TLR8 (dHsaCNS317664208), RELA; nuclear factor NF‐kappa‐B (NF‐κB) p65 (dHsaCNS873554652), autophagy related 7 (ATG7) (dHsaCNS131828358), and gamma‐aminobutyric acid receptor‐associated protein‐like 2 (GABARALPL2) (dHsaCNS699447999), TaqMan Gene Expression Assay (VIC) of HPRT1 (Life Technologies, 4448509), and Bio‐Rad ddPCR reagents/consumables according to the manufacturer's instructions. The percentage of copy number variation of different genes of interest at 2 and 6 months after treatment was quantified relative to the baseline expression.

### 2.7 Western blot analysis

Frozen monocyte samples were thawed on ice, centrifuged to remove the freezing medium, and washed twice with ice‐cold PBS, then total protein was extracted using 200 µl of 1x radioimmunoprecipitation assay (RIPA) lysis buffer (Millipore Sigma, 20–188), supplemented with protease and phosphatase inhibitor cocktail (Millipore Sigma, PPC1010). Protein concentration was determined utilizing Micro BCA Protein Assay Kit (Thermo Fisher Scientific, 23235) following the manufacturer's instructions. Protein lysates (25 µg) were resolved by SDS‐Polyacrylamide gel electrophoresis (PAGE) and transferred to Immobilon‐P polyvinylidene fluoride (PVDF) membrane (Millipore Sigma, IPVH00010). Membranes were blocked in 5% non‐fat milk in Tris‐buffered saline with 0.1% Tween 20 detergent (TBST) buffer at room temperature for 1 h, followed by incubation with primary antibodies to LRRK2 (1:2000, Thermo Fisher Scientific, MA511154), HMOX1 (1:1000, Thermo Fisher Scientific, MA1112), TLR2 (1:1000, Proteintech, 66645‐1‐Ig), TLR8 (1:1000, Origene, TA810175), RELA (1:1000, Thermo Fisher Scientific, 436700), ATG7 (1:1000, Cell Signalling, 8558S), GABARALPL2 (1:1000, Abcam, ab126607), and *β*‐actin (1:3000, Sigma‐Aldrich, A3854) at 4°C overnight, followed by 1 h incubation in 2.5% non‐fat milk in TBST buffer with horseradish peroxidase‐conjugated anti‐rabbit (1:2000, R&D Systems, HAF008) or mouse (1:2000, R&D Systems, HAF018) secondary antibody. Immunoreactive bands were detected using SuperSignal West Pico Chemiluminescent substrate (Thermo Fisher Scientific, 34080), and images were captured using an iBright CL750 Imager (Thermo Fisher Scientific). Immunoblots were quantified using ImageJ software (NIH) relative to *β*‐actin expression. The relative expression of proteins at 2 and 6 months after treatment initiation was quantified relative to baseline levels.

### 2.8 Statistical analysis

Sample size estimates of five PD patients were determined to provide 80% power and to afford an increased score of 1.63 (32%) in baseline immune response using a two‐sided Wilcoxon test assuming normal distribution.^14^ Results are presented as the mean ± standard deviation (SD). Non‐parametric Kruskal–Wallis test with Dunn's multiple test was used to compare the mean ranks of gene/protein expression for all subjects together between different time points. *P* values ≤ 0.05 were considered statistically significant. Statistical analysis was performed using GraphPad Prism 9.1.0 software (GraphPad Software, San Diego, CA). All correlation analyses were determined using Pearson product‐moment correlation coefficients, best‐fit lines were determined using multiple linear regression, *p* values were determined for *r* values greater than 0.25, and multiple *p* values adjusted for FDR at 5%.^28^

## 3 RESULTS

### 3.1 Study population and demographics

In this study, six PD patients were enrolled and five met the study entry criteria. Average age of the patients was 64 ± 5 years, and anti‐parkinsonian therapies (carbidopa‐levodopa) were continued during the study course (Table 1).

**TABLE 1:** Study population demographics (modified from^14^)

	**Number of patients**	**Mean ± SD**
Age (years)	5	64 ± 5
Disease duration since diagnosis	5	8 ± 5
MDS‐UPDRS III score	5	20 ± 5
Sex (male)	5	–
Carbidopa‐Levodopa 25–100 mg^a^	3	–
Carbidopa‐Levodopa 50–200 mg	1	–
Carbidopa‐Levodopa 23–95 mg	1	–

Values are depicted as mean ± SD.

^a^Subject 2001 started anti‐parkinsonian therapy on month 8.

### 3.2 Proteomic and single‐cell RNA sequencing monocyte analyses

Before initiating sargramostim treatment, subjects underwent three UPDRS assessments at monthly intervals to establish baseline motor function for disease progression monitoring. After initiating sargramostim treatment, UPDRS was assessed every 4 weeks for all patients. Over the treatment course, sargramostim resulted in an overall decrease in UPDRS part III (UPDRS III) scores compared to baseline for all subjects.^14^ Although, CNS innate immune microglia are comprehensively studied in PD animal models and patients,^3^, ^29^, ^30^ peripheral monocytes have received less attention due to a past perception of immune privileged CNS. Altered monocyte functions have been reported in the periphery and CSF of PD patients.^3^ To elucidate mechanisms of clinical findings observed in PD patients following sargramostim treatment, we analysed monocyte proteome from cells collected at 2‐ and 6‐months following treatment. Comparisons were adjusted from baseline measures using *Homo sapiens* proteome databases (UniProt: proteome ID: UP000005640, Swiss‐Prot: reviewed proteins). Three patients (2003, 2004, and 2005) with UPDRS III scores 5 points below baseline^14^ were selected and subjected to scRNA‐seq and proteomic analyses at 6 months following treatment. Expression of over 2000 proteins were identified by LFQ at both time points after treatment initiation (Additional file 1). Amongst the total identified proteins, 259 and 879 proteins were differentially expressed (*p* ≤ 0.05) at 2 and 6 months after treatment initiation, respectively (Additional file 1), and 25 and 262 proteins were differentially expressed after adjusting *p* values for FDRs (Additional file 2). Expression of 15 proteins out of 25 proteins overlapped between 2‐ and 6‐months (Additional file 2). Interestingly, 5/15 of the proteins (tryptase beta‐2 [TPSB2], HMOX1, LRRK2, TLR8, and superoxide dismutase 2 [SOD2]) showed significant downregulation at both time points, suggesting the induction of antioxidant, anti‐inflammatory, and autophagy activities by 2 and 6 months after treatment initiation. Volcano plots show differentially expressed proteins at 2 and 6 months after treatment compared to baseline (Figure S1). For scRNA‐seq, the expression of 27,875 genes were identified and quantified using a NextSeq500 sequencer (Additional file 3). Amongst total identified genes, 746 were differentially expressed at 6 months of treatment compared to baseline (*p* ≤ 0.05).

### 3.3 Functional and pathway enrichment of proteomic and scRNA‐seq analyses

**FIGURE 1:**
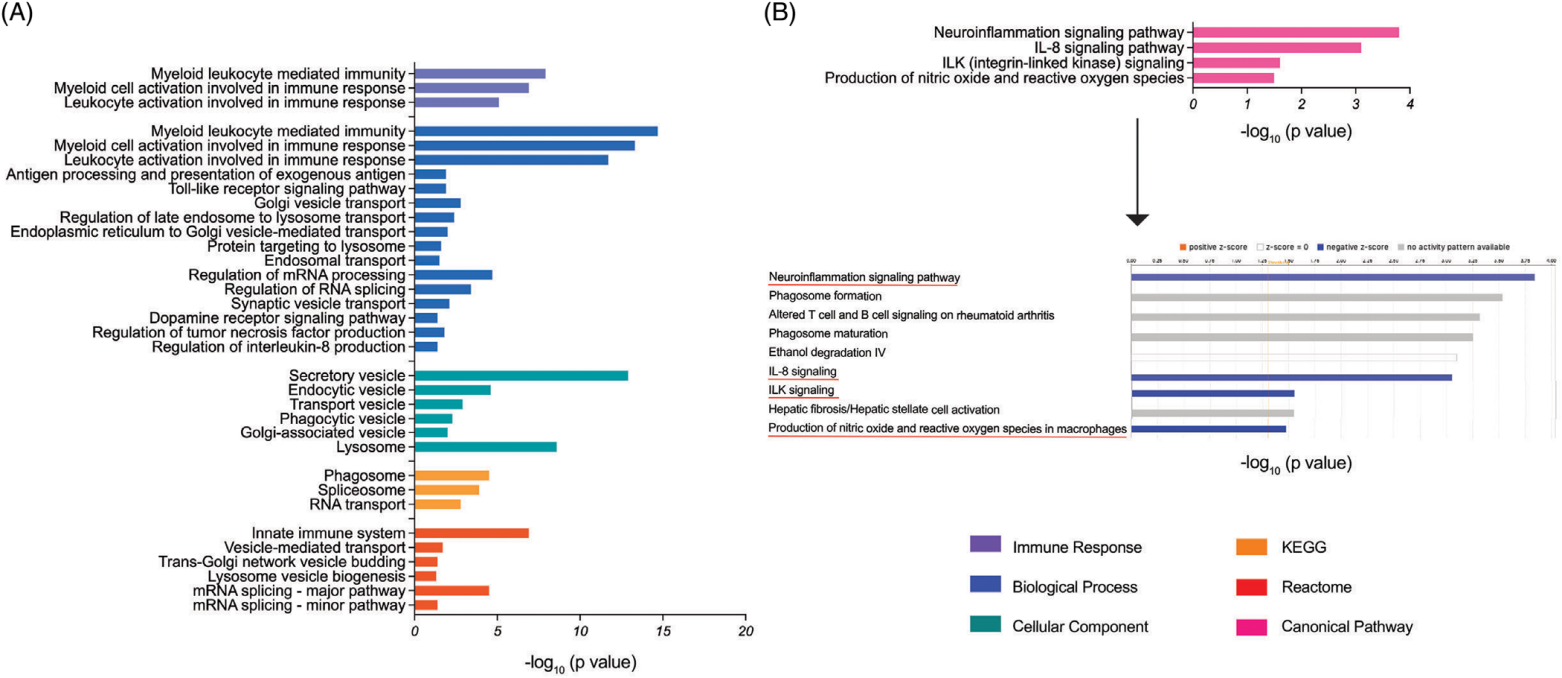
Pathway enrichment of differentially expressed proteins in monocytes of PD patients at 2 months after sargramostim treatment. (A) Gene ontology (GO)‐term functional enrichment by five categories (immune response, biological process, cellular component, KEGG, and Reactome) performed using Cytoscape in conjunction with the plug‐in ClueGO. (B) Canonical pathway enrichment analysis performed using IPA (Qiagen). Black arrow points to the state of canonical pathways illustrated in Figure 1B; orange colour (activation), blue colour (inhibition), and grey colour (no activity pattern)

To understand changes in the monocyte proteomic profile due to sargramostim treatment, we performed functional and pathway enrichment analyses of differentially regulated proteins at 2 and 6 months after initiation of sargramostim. By 2 months, multiple immune processes were found enriched, including “myeloid leukocyte mediated immunity” (*p* = 1.33 × 10^−8^), “myeloid cell activation involved in immune response” (*p* = 1.30 × 10^−7^), and “leukocyte activation involved in immune response” (*p* = 7.25 × 10^−6^) (Figure 1A and Additional file 4). Similarly, KEGG and Reactome analyses showed “phagosome” (*p* = 2.74 × 10^−4^) and “innate immune system” (*p* = 1.29 × 10^−7^) enrichments, respectively. Additionally, pathways involved in regulation of interleukin‐8 (IL‐8) (*p* = 0.0364) and tumour necrosis factor (TNF) production (*p* = 0.0173) were enriched. These data highlight the control of inflammation by sargramostim treatment. Interestingly, Ingenuity Pathway Analysis (IPA) showed inhibition of neuroinflammation signalling (*p* = 1.4 × 10^−4^), IL‐8 (*p* = 8.8 × 10^−4^), integrin‐linked kinase (ILK) (*p* = 0.028), nitric oxide and reactive oxygen species (ROS) (*p* = 0.0335) pathways (Figure 1B and Additional file 5). Additionally, sargramostim affected transport mechanisms associated with endosomes (*p* = 0.0314), Golgi vesicles (*p* = 0.0016), endoplasmic reticulum to Golgi vesicle transfer (*p* = 0.0102), protein targeting to lysosome (*p* = 0.0278), and late endosome to lysosome maturation (*p* = 0.0039) (Figure 1A and Additional file 4). Accordingly, cellular component and Reactome analyses showed enrichment of secretory vesicles (*p* = 1.14 × 10^−13^) endocytic vesicles (*p* = 2.37 × 10^−5^), transport vesicles (*p* = 0.0012), phagocytic vesicles (*p* = 0.0052), Golgi‐associated vesicles (*p* = 0.0092), lysosomes (*p* = 2.59 × 10^−9^), and trans‐Golgi network vesicle budding (*p* = 0.0419) (Figure 1A and Additional file 4). Sargramostim treatment induced changes in biological processes related to the RNA processing such as regulation of mRNA processing (*p* = 2.16 × 10^−5^) and regulation of RNA splicing (*p* = 3.96 × 10^−4^). Similarly, KEGG and Reactome tests showed pathways affecting RNA processing (including RNA transport, *p* = 0.0370), mRNA splicing (major and minor pathways, *p* = 3.5 × 10^−5^ and *p* = 0.0431, respectively), and spliceosome (*p* = 0.0012). These results indicate that homeostatic monocyte control ensued as early as 2 months after initiation of sargramostim treatment.

**FIGURE 2:**
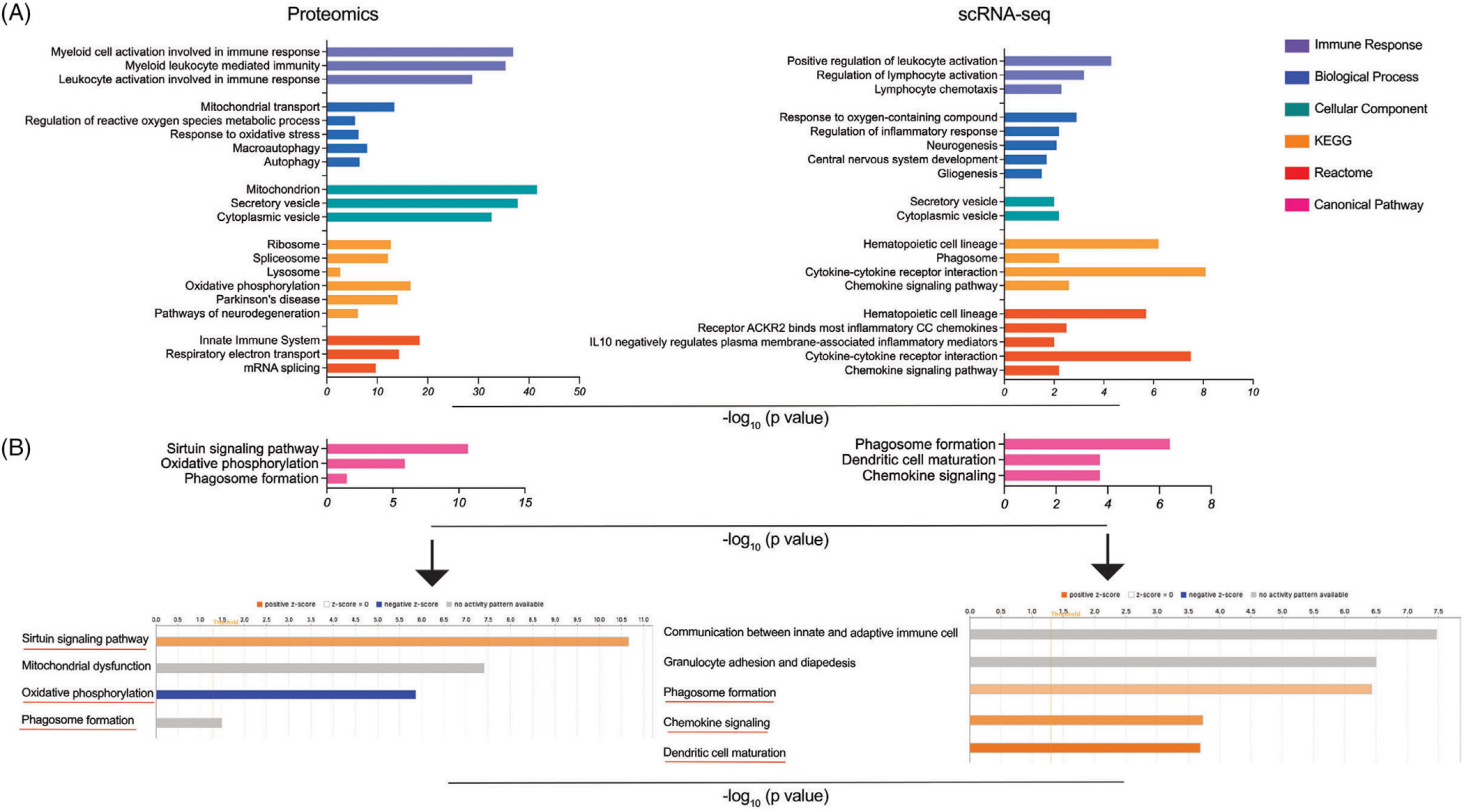
Pathway enrichment of differentially expressed proteins/genes in monocytes at 6 months after sargramostim treatment. (A) GO‐term functional enrichment by five categories (immune response, biological process, cellular component, KEGG, and Reactome) was performed using Cytoscape in conjunction with the plug‐in ClueGO. (B) Canonical pathway enrichment analysis was performed using IPA (Qiagen). Black arrows point to the state of canonical pathways illustrated in Figure (B); orange colour (activation), blue colour (inhibition), and grey colour (no activity pattern)

Both proteomic and scRNA‐seq analyses at 6 months of treatment displayed enrichment of immune processes, including monocyte‐macrophage mediated immunity (*p* = 6.03 × 10^−34^), activation (*p* = 2.29 × 10^−35^), and innate immune responses (*p* = 2.91 × 10^−27^). Moreover, regulation of leukocyte activation (*p* = 0.0050), lymphocyte chemotaxis (*p* = 0.0050), and regulation of lymphocyte activation (*p* = 6.54 × 10^−4^) were shown (Figure 2A and Additional files 6 and 7). Similarly, proteomic Reactome analysis showed enrichment of innate immunity (*p* = 3.66 × 10^−19^) and phagosome formation (*p* = 0.0328) (Figure 2A,B and Additional files 6 and 8). IPA of 6 month‐scRNA‐seq data sets showed chemokine signalling (*p* = 1.84 × 10^−4^), dendritic cell maturation (*p* = 2.03 × 10^−4^), and phagosome formation (*p* = 3.73 × 10^−7^) enrichments (Figure 2A,B and Additional file 9). These data together indicate that sargramostim activated several different immune processes by 6 months of treatment initiation. In addition, there was an enrichment of regulation of ROS metabolism (*p* = 0.0032), oxidative stress (*p* = 7.07 × 10^−4^), and oxygen containing compounds (*p* = 0.0012) (Figure 2A and Additional files 6 and 7). These data suggest antioxidant effects following sargramostim treatment. Interestingly, IPA of 6‐month proteomic data showed inhibition of oxidative phosphorylation (*p* = 1.36 × 10^−6^) (Figure 2B and Additional file 8), suggesting that sargramostim treatment controls ROS production. Moreover, 6‐month proteomic data depicted an enrichment of mitochondria function (*p* = 8.26 × 10^−40^) as the primary organelle responsible for respiratory electron transport pathway enriched in Reactome analysis (*p* = 5.29 × 10^−15^) (Figure 2A and Additional file 6). Additionally, cellular component analysis of both proteomic and scRNA‐seq data resulted in enrichment of secretory (*p* = 1.59 × 10^−38^) and cytoplasmic (*p* = 0.0066) vesicles (Figure 2A and Additional files 6 and 7). These data imply an accelerated bidirectional movement of materials across the cell membrane with sargramostim treatment. Importantly, 6‐month proteomics demonstrated enriched autophagy processes (*p* = 4.63 × 10^−4^) and macroautophagy (*p* = 1.51 × 10^−5^) (biological process analysis) as well as activation of sirtuin signalling pathway (IPA, *p* = 2.19 × 10^−11^) (Figure 2A,B and Additional files 6 and 8), which can regulate autophagy^31^ and serve as an important mechanism for removal of misfolded *α*‐syn. Notably, 6‐month scRNA‐seq data showed enrichment of pathways linked to regulation of inflammatory responses (*p* = 0.0059), “IL‐10 mediated negative regulation of plasma membrane‐associated inflammatory mediators” (*p* = 0.0097),^32^, ^33^ and “receptor ACKR2 binding most inflammatory CC chemokines” (*p* = 0.0032)^34^ (Figure 2A and Additional file 7). These data highlight the anti‐inflammatory responses invoked by 6‐month sargramostim treatment. Overall, the results indicate antioxidant and anti‐inflammatory responses as well as autophagy after sargramostim initiation. The protein‐protein interactions (PPIs) related to five categories (biological processes, cellular components, molecular functions, KEGG pathways, and Reactome pathways) were also identified using STRING analysis of their interaction networks for proteomic data at 2 and 6 months of treatment (Additional files 10 and 11). These data demonstrate a complex immunomodulatory, antioxidant, anti‐inflammatory, and autophagy network interactions.

### 3.4 scRNA‐seq data sets demonstrate an anti‐inflammatory monocyte phenotype induced by sargramostim treatment

For deeper examination of cell heterogeneity, scRNA‐seq data was clustered as cell groupings by measuring gene transcript similarities and differences.^35^ Interestingly, monocyte gene clustering showed a remarkable alteration of transcriptome signatures for patients 2004 and 2005 at 6 months compared to baseline (Figure S2). In addition, scRNA‐seq clustering was examined to assess putative monocyte phenotypes by measuring gene expression of *CD14*, *CD16*, *CD93*, *CD163*, and *CD209*, all associated with monocyte surface markers, at baseline and 6 months after sargramostim was started. *CD14*, was expressed in 82% and 70% of cells at baseline and 6 months, respectively, while no differences in *CD16* expression were detected. This suggests that most of the monocytes assessed by scRNA‐seq at both time points exhibited a classical monocyte phenotype (CD14^+^CD16^−^). In addition, *CD93* and *CD163* genes encoding anti‐inflammatory surface markers,^36^ were expressed by 66% and 44%, respectively, at 6 months compared to 43% and 31%, respectively, at baseline. Notably, expression of *CD209* encoding for inflammatory monocyte surface marker,^37^ was not changed compared to baseline. Together, these observations demonstrate an anti‐inflammatory transcriptomic phenotype by monocytes of sargramostim‐treated PD patients.

### 3.5 Sargramostim induces monocyte antioxidant, anti‐inflammatory, and autophagy‐related activities

Bioinformatic analyses identified proteins with antioxidant, anti‐inflammatory, and autophagy functions that were differentially expressed by 2 and 6 months of sargramostim treatment (Table 2). Antioxidant and anti‐inflammatory proteins included HMOX1, TLR2, TLR8, and RELA all were downregulated at 2 months of treatment. Autophagy‐inducing proteins included ATG3, ATG7, and GABARAPL2; all were upregulated at 6 months of sargramostim treatment. Downregulation of HMOX1 by 2 months (–2.5‐fold, *p* = 3.47 × 10^−8^) and 6 months (–3.1‐fold, *p* = 9.58 × 10^−12^) of treatment supports an antioxidant effect of sargramostim on therapeutic outcomes for PD. In addition, downregulation of TLR2 and TLR8 by 2 months (−1.77‐fold, *p* = 0.0348; and −1.99‐fold, *p* = 3.09 × 10^−6^, respectively) and 6 months (−1.66‐fold, *p* = 1.06 × 10^−6^; and −2.598‐fold, *p* = 1.66 × 10^−6^, respectively) of treatment supports an anti‐inflammatory neuroprotective signature for sargramostim therapy in PD. Moreover, our data showed significant downregulation of RELA and IKBKG by 2 months (−2.74‐fold, *p* = 0.0147; and −1.89‐fold, *p* = 0.0138, respectively) of treatment, thus provided further support for an anti‐inflammatory mechanism of sargramostim.

**TABLE 2:** Changes in antioxidant, anti‐inflammatory, and autophagy proteins after sargramostim treatment

**Months after treatment initiation**	**Canonical pathway**	**Direction**	***p* value**	**Differentially expressed proteins(Fold change, *p* value)**
**2 months**	Neuroinflammation signalling pathway	Inhibition	1.43E‐04	HMOX1 (−2.49, 3.47E‐08) TLR2 (−1.77, 3.48E‐02) TLR8 (−1.99, 3.09E‐06) IKBKG (−1.89, 1.38E‐02)
	IL‐8 signalling pathway	Inhibition	8.8E‐04	IKBKG (−1.89, 1.38E‐02) HMOX1 (−2.49, 3.47E‐08)
	Production of nitric oxide and reactive oxygen species	Inhibition	3.35E‐02	IKBKG (−1.89, 1.38E‐02) TLR2 (−1.77, 3.48E‐02)
	Integrin‐Linked Kinase (ILK) signalling pathway	Inhibition	2.8E‐02	RELA (−2.74, 1.47E‐02)
**6 months**	Sirtuin signalling pathway	Activation	2.19E‐11	ATG3 (1.55, 2.79E‐02) ATG7 (1.68, 4.28E‐02) GABARAPL2 (3.24, 9.57E‐03)
	Oxidative phosphorylation	Inhibition	1.36E‐06	ATP5F1D (−1.856, 1.06E‐05) ATP5PB (−1.638, 1.96E‐06) ATP5PF (−1.719, 6.24E‐06) ATP5PO (−1.573, 1.44E‐04) COX5B (−1.736, 7.79E‐09) COX7C (−1.839, 8.2E‐07) NDUFA2 (−1.902, 2.52E‐05) NDUFB1 (−1.561, 3.06E‐02) NDUFB4 (−1.994, 1.13E‐03) NDUFS2 (−1.87, 1.34E‐04) NDUFS3 (−1.835, 3.03E‐07) NDUFS6 (−29.314, 1.66E‐03) NDUFS7 (−1.535, 8.02E‐06) SDHA (−1.563, 1.71E‐08)

A defective chaperone‐mediated autophagy (CMA) pathway underlies PD‐associated neurotoxicity by compromising *α*‐syn degradation. Thus, significant downregulation of LRRK2 after 2 months (−2.10‐fold, *p* = 8.54 × 10^−6^) and 6 months (−1.71‐fold, *p* = 9.38 × 10^−4^) of treatment suggests an autophagy‐inducing mechanism where sargramostim provides protective outcomes in PD. Moreover, several autophagy‐related proteins such as ATG3, ATG7, and GABARAPL2 regulate different autophagy signalling pathways.^38^, ^39^, ^40^ Therefore, the significant upregulation of ATG3, ATG7, and GABARAPL2 (1.55‐fold, *p* = 0.0279; 1.68‐fold, *p* = 0.0428; and 3.24‐fold, *p* = 0.0096, respectively) after 6 months of treatment indicates increased autophagy‐inducing functions by sargramostim providing a protective outcome in PD patients. Based on these observations, we selected LRRK2, HMOX1, TLR2, TLR8, RELA, ATG7, and GABARAPL2 for measurement by ddPCR and Western blot analyses to assess the feasibility of using these genes/proteins as reliable biomarkers for detecting patient responses to sargramostim treatment with beneficial outcomes.

### 3.6 The ddPCR and protein analyses of potential PD biomarkers

**FIGURE 3:**
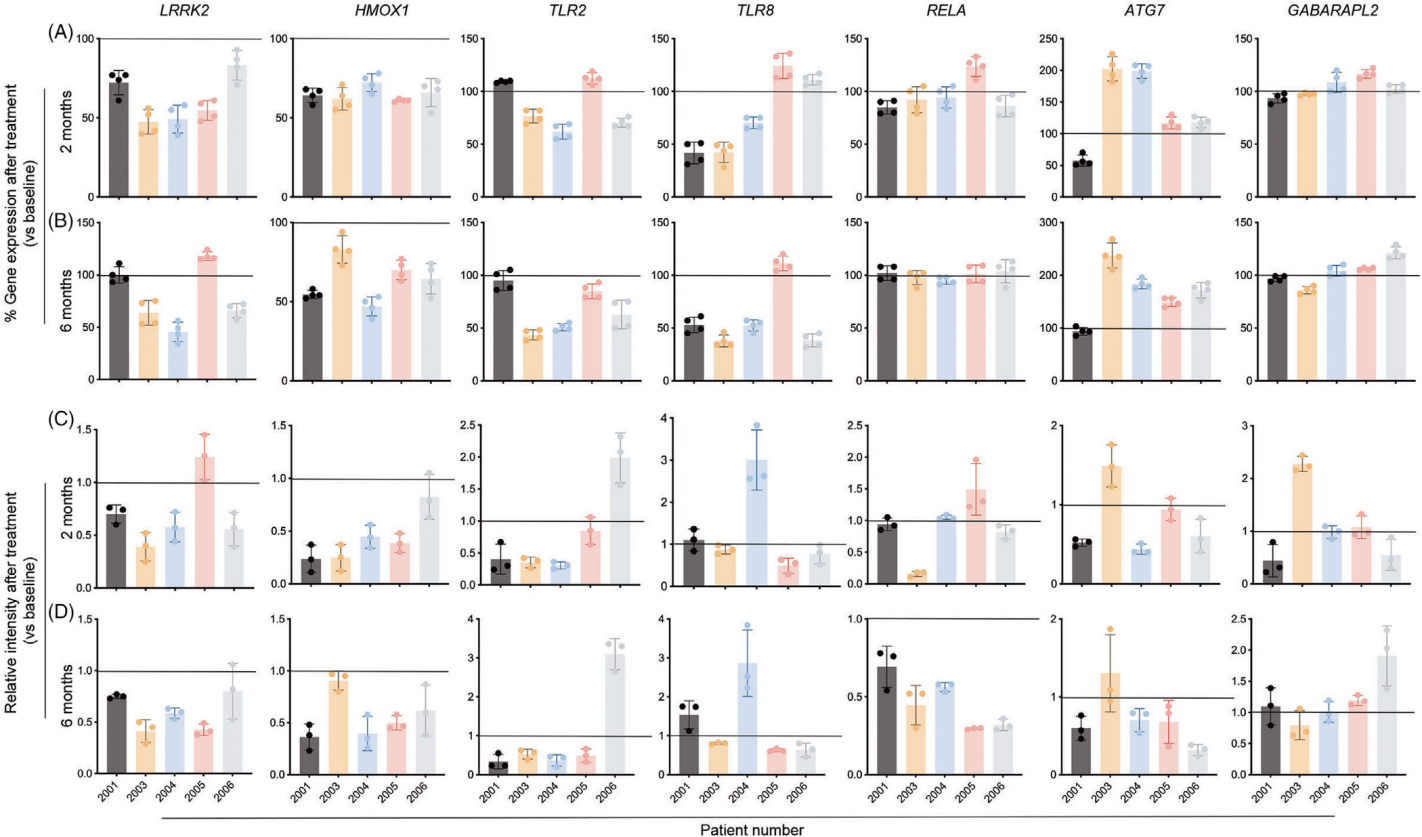
Gene and protein expression of potential biomarkers in monocytes at 2 and 6 months after sargramostim treatment. The ddPCR assay was performed to determine the gene expression of *LRRK2, HMOX1, TLR2, TLR8, RELA, ATG7*, and *GABARAPL2* at 2 (A) and 6 (B) months after starting the sargramostim treatment compared to baseline. Gene expression was normalized to *HPRT1*, and the ddPCR assay was performed four times (*n* = 4 technical replicates). Western blot analysis was performed to determine the protein expression of *β*‐actin, LRRK2, HMOX1, TLR2, TLR8, RELA, ATG7, and GABARAPL2 at 2 (C) and 6 (D) months after starting the sargramostim treatment compared to baseline. Protein expression was normalized to *β*‐actin and densitometric quantification is shown. Western blot analysis was done thrice (*n* = 3 technical replicates). Data represent mean ± SD. Horizontal line in each image represents baseline expression; values above the line indicate upregulation, while values below the line indicate downregulation

Western blot and ddPCR analyses affirm the expression of putative proteins and genes, respectively. These are shown in Figure S3. The variability in therapeutic responses is acknowledged between individual subjects. In attempts to address this and the sample size limitation we compared gene expression of a group of biomarkers during study and adjusted to baseline measurements. The analyses included *LRRK2*, *HMOX1*, *TLR2*, *TLR8*, *RELA*, *ATG7*, and *GABARAPL2* examined for baseline measures and at 2 and 6 months after initiating sargramostim. At 2 months after initiation of therapy, gene expression was decreased for *LRRK2* and *HMOX1* in all subjects and decreased *TLR2* and *TLR8* was detected in 3 subjects (Figure 3A). Four of five subjects demonstrated decreased *RELA* gene expression, but was limited to 15% of baseline. For *ATG7* gene 4/5 of subjects showed upregulation but at or below 17% of baseline measures in two subjects (2005 and 2006). Similarly, for *GABARAPL2* gene 3/5 subjects showed upregulation but at or below 17% of baseline measurements. At 6 months, gene expression was decreased for *HMOX1* and *TLR2* in all patients, and for *LRRK2* and *TLR8* by 3/5 and 4/5 of patients, respectively (Figure 3B). *RELA* was downregulated by 2/5, but did not exceed 5% of baseline. Similar to 2‐month ddPCR results, *ATG7* and *GABARAPL2* were found to be upregulated in 4/5 and 3/5 of tested subjects, but failed to exceed 6% of baseline for *GABARAPL2*. Similarly, we compared protein expression of LRRK2, HMOX1, TLR2, TLR8, RELA, ATG7, and GABARAPL2 at baseline, and at 2 and 6 months of sargramostim treatment. At 2 months, 4/5 of patients exhibited decreased LRRK2 and HMOX1, while TLR2 was diminished in 3/5 of subjects (Figure 3C). In addition, 3/5 of subjects showed decreased protein expression of TLR8 and RELA. Additionally, one subject displayed increased expression of ATG7 and GABARAPL2 proteins. At 6 months, 4/5 of subjects showed TLR2 downregulation (Figure 3D). Notably, LRRK2, HMOX1, TLR8 and RELA were reduced in most subjects. One subject displayed increased expression of ATG7, while GABARAPL2 was upregulated in 3/5 of subjects.

In addition, for all subjects we compared relationships between gene and protein expression of LRRK2, HMOX1, TLR2, TLR8, RELA, ATG7, and GABARAPL2 (Table 3 and Figure S4A,B). There was a significant difference in gene expression of *LRRK2*, *HMOX1*, and *TLR2* (*p* = 0.0213, 0.0021, and 0.0452, respectively). The gene expression of *LRKK2* and *HMOX1* decreased significantly at 2 months of treatment (*p* = .0286 for both genes). In addition, at 6 months the expression of *HMOX1* decreased significantly (*p* = 0.0149), while the downregulation of *TLR2* gene at the level of significance (*p* = 0.0525). At the protein level, there was significant difference in gene expression of *LRRK2*, *HMOX1*, and *RELA* at different time points (*p* = 0.0345, 0.0007, and 0.0257, respectively). The protein levels of HMOX1 and RELA significantly decreased (*p* = 0.0059 and 0.0352, respectively) after 2 and 6 months of treatment, respectively. While at 6 months of treatment, the protein levels of LRRK2 and HMOX1 were decreased but not significantly (*p* = 0.0636 for both proteins). Together, these results support the bioinformatic analyses that demonstrated antioxidant, anti‐inflammatory, and autophagy treatment effects in PD monocytes.

**TABLE 3:** Differences in the mean values of gene/protein expression during sargramostim treatment

	**ddPCR**						
	***LRRK2***	***HMOX1***	***TLR2***	***TLR8***	***RELA***	***ATG7***	***GABARAPL2***
**Kruskal–Wallis test (*p* value)**	0.0213	0.0021	0.0452	0.3009	.1644	.1655	.8528
	**Dunn's multiple comparison test (adjusted *p* value)**						
2M versus baseline 6M versus baseline 6M versus 2M	.0286	0.0286	>.9999	>.9999	0.4495	.4495	>.9999
	.2519	.0149	.0525	0.3395	>.9999	0.2155	>0.9999
	>.9999	>.9999	.3915	.9402	0.2155	>0.9999	>.9999

	**Western blot**						
	**LRRK2**	**HMOX1**	**TLR2**	**TLR8**	**RELA**	**ATG7**	**GABARAPL2**
**Kruskal‐Wallis test (*p* value)**	0.0345	.0007	0.1796	0.8374	.0257	.1853	0.3515
	**Dunn's multiple comparison test (adjusted *p* value)**						
2M versus baseline 6M versus baseline 6M versus 2M	0.1313	0.0059	0.2519	>.9999	>0.9999	.3146	>.9999
	0.0636	0.0636	0.3915	>0.9999	.0352	0.3146	0.6627
	>0.9999	>0.9999	>0.9999	>0.9999	.2155	>0.9999	0.5138

### 3.7 Integrated scRNA‐seq and proteomic analysis during sargramostim treatment

The overlapping expression of genes and proteins as detected by scRNA‐seq and proteomic analyses for subjects 2003, 2004, and 2005 (Additional files 3 and 12, respectively) were identified and illustrated in Additional file 13. Notably, most of the proteins identified in the proteomic analysis (2,507 out of 2,560 genes) have been identified in scRNA‐seq analysis as well. Venn diagram depicts the number of genes identified in each data set and the number of overlapped genes in both data sets (Figure S5A). In addition, the unique genes in each data set were identified and illustrated in Additional file 14. Pearson product‐moment correlation coefficient between scRNA‐seq and proteomic overlapping genes has been calculated (*r* = 0.3582, *p* = 8.9627 × 10^−77^), which showed significant moderate positive association between mRNA and protein expressions (Figure S5B). This indicates that expression of multiple genes was altered in the same direction, either down‐ or up‐regulated.

### 3.8 Western blot and ddPCR UPDRS III score correlations

**FIGURE 4:**
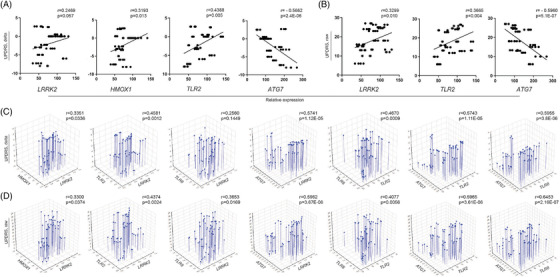
Prediction of UPDRS III score by gene expression of potential biomarkers. (A) Correlation between genetic expression of *LRRK2*, *HMOX1*, *TLR2*, and *ATG7* and change in UPDRS III score. (B) Correlation between genetic expression of *LRRK2*, *TLR2*, and *ATG7* and raw UPDRS III score. (C) Multiple linear regression analysis of effect of genetic expression of *LRRK2*, *HMOX1*, *TLR2*, *TLR8*, and *ATG7* on change in UPDRS III score. (D) Multiple linear regression analysis of effect of genetic expression of *LRRK2*, *HMOX1*, *TLR2*, *TLR8*, and *ATG7* on raw UPDRS III score. (A,B) *r* = Pearson product‐moment correlation coefficient. (C,D) *r* = regression coefficient. *p* ≤ 0.05 was considered significant

Our study significantly adds to the knowledge of gene‐protein therapeutic response signatures of monocytes from PD patients during treatment with sargramostim. Correlating the expression of potential gene or protein biomarkers with UPDRS III scores would benefit in identifying the capacity to assess disease progression and predict putative therapeutic response for immunomodulatory drugs such as sargramostim. Therefore, we performed correlation analysis of UPDRS III scores or its change from baseline with gene expression of selected biomarkers (Table 4), including *LRRK2* (*r* = 0.2469, *p* = 0.057), *HMOX1* (*r* = 0.3193, p = 0.013) and *TLR2* (*r* = 0.4388, *p* = 0.005) (Figure 4A). Each demonstrated relationships between decreased gene expression and improved motor function. An indirect correlation was shown between the change in UPDRS III scores and *ATG7* expression (*r* = −0.5662, *p* = 2.4 × 10^−6^) (Figure 4A). Similarly, total UPDRS III scores were directly correlated with expression of *LRRK2* (*r* = 0.3299, *p* = 0.01) and *TLR2* (*r* = 0.3665, *p* = 0.004), while indirectly correlated with *ATG7* (*r* = −0.5960, *p* = 5.1 × 10^−7^) (Figure 4B). We next assessed the predictive potential for those correlated pairs and changes in UPDRS III scores from baseline and total UPDRS III scores by multiple regression analysis (Tables 5 and 6, respectively). *LRRK2* and *HMOX1* showed significant effect on UPDRS III score change (*r* = 0.3539, *p* = 0.0336) (Figure 4C) with *HMOX1* having a stronger, though not significant effect (*β* = 0.2611, *p* = 0.0747), whereby change in scores would increase by 4.1 points per fold increase in ddPCR *HMOX1* expression (Table 5). The effect of *LRRK2* and *TLR2* was significant (*r* = 0.4581, *p* = 0.0012) (Figure 4C), however *TLR2* provided a greater effect (*β* = 0.4013, *p* = 0.0018) predicting an increase of 5 points in change from baseline scores per unit increase in *TLR2* expression (Table 5). *LRRK2* and *ATG7* showed strong potential to affect changes in UPDRS III score (*r* = 0.5741, *p* = 1.12 × 10^−5^) (Figure 4C) while *ATG7* provided a greater effect (*β* = −0.6326, *p* = 1.3 × 10^−6^) and would predict a lower UPDRS score from baseline score by 4 points per fold increase in expression of *ATG7* (Table 4). The combined effect of *TLR2* and *TLR8* was significant (*r* = 0.4671, *p* = 0.0009) (Figure 4C) with *TLR2* showing a greater effect (*β* = 0.5124, *p* = 0.0002) and increasing the change in baseline scores by 6.4 points per unit increase in *TLR2* gene expression (Table 5). The combination of *TLR2* and *ATG7* expression was significantly linked to the UPDRS III score change (*r* = 0.5743, *p* = 1.1 × 10^−5^) (Figure 4C), with *ATG7* having the greater influence (*β* = −0.4850, *p* = 0.0012) providing 3.1 points decrease in change in baseline score per unit increase in *ATG7* expression (Table 5). The combined effect of *TLR8* and *ATG7* correlated with the change in UPDRS score (*r* = 0.5955, *p* = 3.8 × 10^−6^) (Figure 4C) with *ATG7* producing a greater diminution effect (*β* = −0.6398, *p* = 6.8 × 10^−7^) whereby a 1‐fold increase in *ATG7* expression decreased UPDRS III score change by 4 points (Table 5). Interestingly, the combined effects of all 7 variables showed a strong correlation on score changes from baseline (*r* = 0.6904, *p* = 1.0 × 10^−5^) that accounted for 48% the variation in change from baseline (Table 5). The 3 variables producing the strongest effects from baseline scores, *HMOX1*, *TLR8*, and *ATG7*, were significantly correlation (*r* = 0.6354, *p* = 2.0 × 10^−6^) and accounted for 40% of the score change from baseline (Table 5). The strongest effect on change from UPDRS baseline was provided by *ATG7* (*β* = −0.5973, *p* = 2.1 × 10^−6^) leading to a 3.8‐point diminution per fold increase in gene expression.

**TABLE 4:** Correlation of UPDRS III scores and ddPCR relative expression

		UPDRS, Total	**UPDRS, Change**	***LRRK2***	***HMOX1***	***TLR2***	***TLR8***	***RELA***	***ATG7***	***GABARAPL2***
UPDRS, Total	*r*	1.0000	0.6867	0.3299	.1598	.3665	−0.0089	−0.1380	−0.5960	−0.0115
	*p*		1.4E‐09	0.0100	0.2226	0.0040	0.9462	0.2931	5.1E‐07	0.9305
UPDRS, Change	*r*	0.6867	1.0000	0.2469	0.3193	0.4388	0.0385	−0.1464	−0.5662	−0.0360
	*p*	1.4E‐09		0.0572	0.0129	.0005	0.7702	0.2643	2.4E‐06	0.7847
*LRRK2*	*r*	0.3299	.2469	1.0000	0.4972	0.2741	0.4069	−0.0481	−0.5733	−.1201
	*p*	0.0100	0.0572		0.0001	0.0341	0.0013	0.7152	1.7E‐06	0.3608
*HMOX1*	*r*	0.1598	0.3193	0.4972	1.0000	0.2171	0.2956	0.0207	−0.2649	−0.1893
	*p*	0.2226	0.0129	0.0001		0.0956	0.0218	0.8750	0.0408	0.1475
*TLR2*	*r*	0.3665	.4388	0.2741	0.2171	1.0000	0.4184	0.1560	−0.6452	−0.0876
	*p*	0.0040	0.0005	0.0341	0.0956		0.0009	0.2340	2.6E‐08	0.5057
*TLR8*	*r*	−0.0089	0.0385	0.4069	0.2956	0.4184	1.0000	0.1341	−0.3704	0.1620
	*p*	0.9462	0.7702	0.0013	0.0218	0.0009		0.3069	0.0036	0.2162
*RELA*	*r*	−0.1380	−0.1464	−0.0481	0.0207	0.1560	0.1341	1.0000	0.0132	0.2511
	*p*	0.2931	0.2643	0.7152	0.8750	0.2340	0.3069		0.9204	0.0530
*ATG7*	*r*	−0.5960	−0.5662	−0.5733	−0.2649	−0.6452	−0.3704	0.0132	1.0000	0.0417
	*p*	5.1E‐07	2.4E‐06	1.7E‐06	0.0408	2.6E‐08	0.0036	0.9204		0.7519
*GABARAPL2*	*r*	−0.0115	−0.0360	−0.1201	−0.1893	−0.0876	0.1620	0.2511	0.0417	1.0000
	*p*	0.9305	0.7847	0.3608	0.1475	0.5057	0.2162	0.0530	0.7519	

*r*, Pearson product moment correlation coefficient; *p*, *p* value.

**TABLE 5:** Multiple linear regression analysis for ddPCR test results on changes in MDS‐UPDRS III scores

**Model**					**Predictors**		
**Predictors**	***r***	***r^2^***	**adj *r^2^***	***p***	***Β***	**b**	***P***
*LRRK2*	0.3351	0.1123	0.0811	0.0336	.1170	1.4967	0.4192
*HMOX1*					0.2611	4.0892	0.0747
*LRRK2*	0.4581	0.2099	0.1822	0.0012	0.1369	1.7505	0.2682
*TLR2*					0.4013	5.0326	0.0018
*LRRK2*	0.2560	0.0655	0.0328	0.1449	0.2771	3.5435	0.0529
*TLR8*					−0.0742	−0.6706	0.5984
*LRRK2*	0.5741	0.3296	.3061	1.1E‐05	−0.1158	−1.4811	0.3853
*ATG7*					−0.6326	−4.0035	1.3E‐05
*TLR2*	0.4670	0.2181	.1906	0.0009	0.5124	6.4261	0.0002
*TLR8*					−0.1759	−1.5889	0.1779
*TLR2*	0.5743	0.3299	0.3063	1.1E‐05	0.1259	1.5786	0.3788
*ATG7*					−0.4850	−3.0693	.0012
*TLR8*	0.5955	0.3546	0.3320	3.8E‐06	−0.1985	−1.7928	0.0886
*ATG7*					−0.6398	−4.0486	6.8E‐07
*LRRK2*	0.6904	0.4767	0.4063	1.0E‐05	−0.1575	−2.0139	0.2809
*HMOX1*					0.3212	5.0298	.0095
*TLR2*					0.2288	2.8699	0.1226
*TLR8*					−0.2830	−2.5557	0.0256
*RELA*					−0.1867	−4.5497	0.0873
*GABARAPL2*					0.1408	4.2734	0.2106
*ATG7*					−0.5320	−3.3667	0.0015
*HMOX1*	0.6354	0.4037	.3718	2.0E‐06	0.2357	3.6915	0.0360
*TLR8*					−0.2524	−2.2801	0.0307
*ATG7*					−0.5973	−3.7800	2.1E‐06

*r*, regression coefficient; *p*, *p* value; *β*, standardized coefficient; b, non‐standardized coefficient.

**TABLE 6:** Multiple linear regression analysis for ddPCR test results on raw MDS‐UPDRS III scores

**Model**				**Predictors**			
**Predictors**	***r***	***r^2^***	**adj *r^2^***	***p***	***β***	**B**	***p***
*LRRK2*	.3300	0.1089	0.0776	0.0374	0.3327	8.2686	.0246
*HMOX1*					−.0056	−0.1711	0.9690
*LRRK2*	0.4374	0.1913	0.1629	0.0024	.2481	6.1659	0.0499
*TLR2*					.2985	7.2750	0.0192
*LRRK2*	0.3653	0.1334	0.1030	0.0169	0.3997	9.9341	0.0045
*TLR8*					−0.1716	−3.0113	0.2089
*LRRK2*	0.5962	0.3554	0.3328	3.7E‐06	−0.0175	−0.4350	0.8932
*ATG7*					−0.6060	−7.4527	1.9E‐05
*TLR2*	0.4077	0.1663	0.1370	0.0056	0.4488	10.9379	0.0014
*TLR8*					−0.1967	−3.4526	0.1451
*TLR2*	0.5965	0.3558	0.3332	3.6E‐06	−0.0309	−0.7524	0.8252
*ATG7*					−0.6159	−7.5743	4.4E‐05
*TLR8*	.6453	0.4164	0.3959	2.2E‐07	−0.2662	−4.6725	0.0177
*ATG7*					−0.6946	−8.5420	3.5E‐08
*LRRK2*	0.6692	0.4478	0.3734	3.6E‐05	0.0727	1.8056	0.6266
*HMOX1*					0.0681	2.0720	0.5807
*TLR2*					0.1300	3.1682	0.3894
*TLR8*					−0.3305	−5.8013	0.0117
*RELA*					−0.1378	−6.5237	0.2162
*GABARAPL2*					0.1338	7.8883	0.2465
*ATG7*					−0.5786	−7.1156	0.0009

*r*, regression coefficient; *p, p* value; *β*, standardized coefficient; b, non‐standardized coefficient.

We also examined whether expression of the same set of genes had effects on total UPDRS III scores (Table 6). In combination, expression of all 7 genes accounted for 67% of the change in total UPDRS scores (*r* = 0.6692, *p* = 3.6 × 10^−5^) from which expression of *ATG7* yielded the most significant of the 7 genes, predicting a diminution of 7.1 points in UPDRS III scores per fold increase in *ATG7* expression (Table 6). Interestingly, 4 combinatorial pairs of genes also demonstrated strong correlations. The correlation of *LRRK2* and *TLR2* combination (*r* = 0.4374, *p* = 0.0024) (Figure 4D) both predicted effects to increase total UPDRS scores by 6.2 and 7.3 points, respectively per fold increase in gene expressions (Table 6). Additionally, a 1‐fold increase in *LRRK2* expression in combination with *HMOX1* or *TLR8* (Figure 4D) was predicted to increase total UPDRS scores by 8.3 and 9.9 points, respectively (Table 6). Contrastingly, while the combination of *LRRK2* and *ATG7* expression strongly correlated with total UPDRS scores (*r* = 0.5962, *p* = 3.7 × 10^−6^) (Figure 4D), *ATG7* exerted a stronger indirect effect on scores (*β* = −0.6060, *p* = 1.9 × 10^−5^) than *LRRK2* predicting a 7.5‐point decrease in total UPDRS scores per fold increase in expression of *ATG7* (Table 6). *ATG7* expression in combination with *TLR2* also predicted a strong influence on total UPDRS scores (*r* = 0.5965, *p* = 3.6 × 10^−6^) (Figure 4D) that would decrease scores by 7.6 points per fold increase in *ATG7* expression (*p* = 4.4 × 10^−5^) (Table 6). The most notable diminutive effect on total scores was afforded by expression of *ATG7* in combination with *TLR8* (*r* = 0.6453, *p* = 2.2 × 10^−7^) (Figure 4D), which predicted a decrease of 8.5 points in total UPDRS scores per fold increase in *ATG7* expression (Table 6). Notably, expression of *TLR2* in combination with *TLR8* (*r* = 0.4077, *p* = 0.0056) (Figure 4D), was determined to have the largest effect on total UPDRS scores with the prediction of adding 10.0 points per fold increase in *TLR2* expression (Table 6).

**FIGURE 5:**
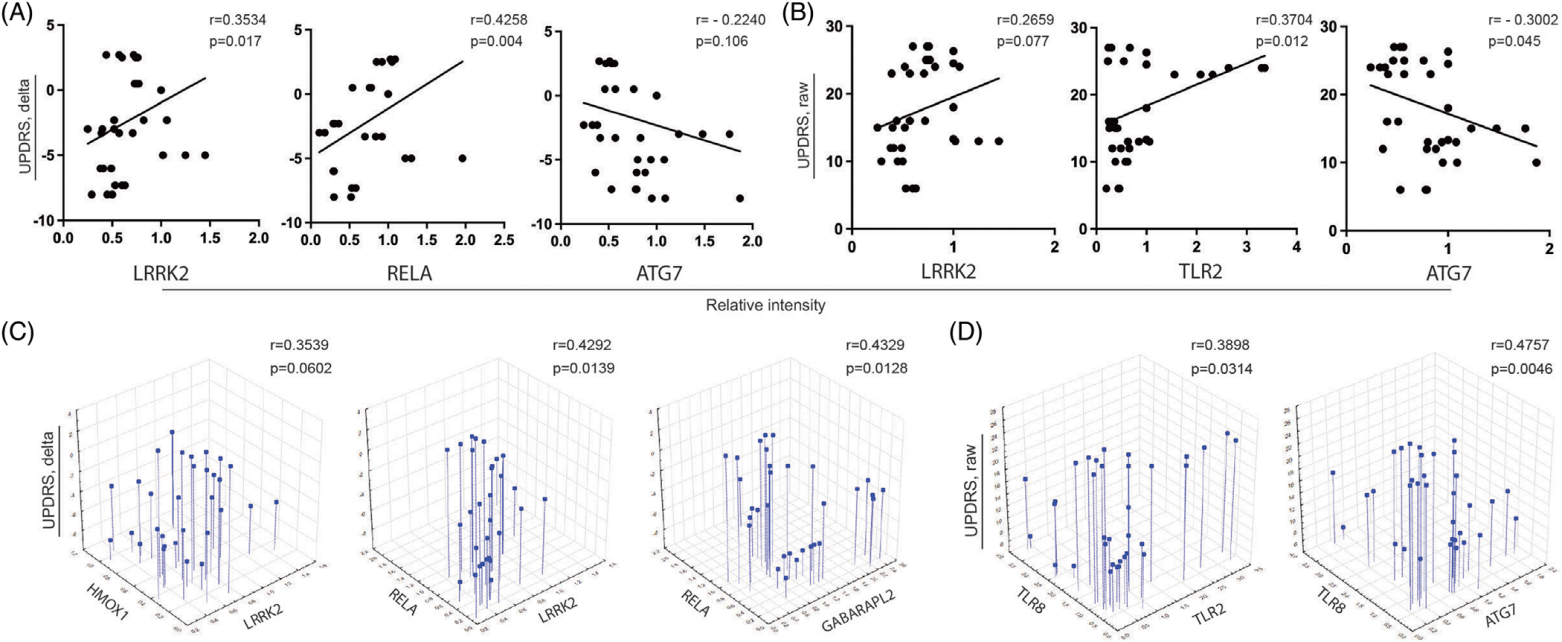
Prediction of UPDRS III score by protein expression of potential biomarkers. (A) Correlation between protein expression of LRRK2, RELA, and ATG7 and change in UPDRS III score. (B) Correlation between protein expression of LRRK2, TLR2, and ATG7 and raw UPDRS III score. (C) Multiple linear regression analysis of effect of protein expression of LRRK2, HMOX1, RELA, and GABARAPL2 on change in UPDRS III score. (D) Multiple linear regression analysis of effect of protein expression of TLR2, TLR8, and ATG7 on raw UPDRS III score. (A,B) *r* = Pearson product‐moment correlation coefficient. (C,D) *r* = regression coefficient. *p* ≤ 0.05 was considered significant

Similarly, changes in UPDRS III scores from baseline were correlated with protein expression of selected biomarkers (Table 7). Positive correlations were shown between change from baseline in UPDRS III scores and protein levels of LRRK2 (*r* = 0.3534, *p* = 0.017) and RELA (*r* = 0.4258, *p* = 0.004) (Figure 5A), suggesting that motor function is improved with decreased expression of these proteins. In addition, an indirect correlation was shown between changes in UPDRS III score and protein level of ATG7 but it did not reach a significant level (*r* = −0.2440, *p* = 0.106). Similarly, positive correlations were shown between total UPDRS III scores and protein levels of LRRK2 (*r* = 0.2659, *p* = 0.077) and TLR2 (*r* = 0.3704, *p* = 0.012), while a negative correlation was shown between scores and protein levels of ATG7 (*r* = −0.3002, *p* = 0.045) (Figure 5B). We next evaluated the predictive value of correlations between Western blot test results (Table 7) and changes from baseline of UPDRS III scores by multiple linear regression analysis (Table 8). The ability of LRRK2 and HMOX1 to predict a change in UPDRS III score trended toward significance (*p* = 0.0602) (Figure 5C). In this model LRRK2 provided a stronger effect (*β* = 0.3594, *p* = 0.0222) with each unit increase in Western blot intensity increasing the change from baseline of UPDRS III scores by 4.3 points (Table 8). The effect of LRRK2 and RELA was significant (*p* = 0.0139) (Figure 5C) with RELA providing a stronger effect (*β* = 0.3655, *p* = 0.0879), although it did not reach the level of significance. The model predicts that the change from baseline of UPDRS III score will increase by 3.3 points/unit increase in NF‐κB p65 Western blot intensity (Table S7). RELA and GABARAPL2 showed significant correlation with UPDRS III score (*r* = 0.4329, *p* = 0.0128) (Figure 5C), and RELA provided a greater effect on the change in UPDRS III score (*β* = 0.4692, *p* = 0.0052) predicting that total UPDRS III scores would increase by 4.2 points per unit increase in RELA intensity (Table 8). The correlated pairs TLR2/TLR8 (*r* = −0.375, *p* = 0.0111) and TLR8/ATG7 (*r* = −0.325, *p* = 0.0294) were assessed as putative predictors of change in UPDRS scores from baseline, however neither pair proved of predictive value (*p* = 0.537 and *p* = 0.2403, respectively). Assessing all 7 Western blot intensities for an effect on change from baseline in UPDRS scores showed moderate correlation that trended to significance (*r* = 0.5359, *p* = 0.0644), yet none proved to have a significant effect on changes in UPDRS scores. However, assessing 3 of the strongest variables, RELA, ATGg7, and GABARAPL2, showed significant correlation (*r* = 0.5007, *p* = 0.0075) with change from baseline UPDRS scores. The stronger effects in this model were exerted by RELA (*β* = 0.4919, *p* = 0.0029) and ATG7 (*β* = −0.2591, *p* = 0.0701), whereby one unit increase in Western blot intensity for RELA or ATG7 would predict a 4.41 increase or a 2.44 decrease in baseline change of UPDRS scores, respectively. In a separate model, RELA and ATG7 correlated with change in scores (*r* = 0.4819, *p* = 0.0039) with RELA providing the stronger effect on change in UPDRS scores (*β* = 0.4159, *p* = 0.0037), whereby change in UPDRS scores would rise by 3.73 points per unit increase in RELA Western blot intensity.

**TABLE 7:** Correlations between UPDRS III and Western blot test scores

		**UPDRS, Total**	**UPDRS, Change**	**LRRK2**	**HMOX1**	**TLR2**	**TLR8**	**RELA**	**ATG7**	**GABARAPL2**
UPDRS, Total	*r*	1.0000	0.6867	0.2659	0.1109	0.3704	−0.2513	0.1847	−0.3002	−0.1110
	*p*		1.9E‐07	0.0774	0.4682	0.0123	0.0958	0.2245	0.0451	0.4681
UPDRS, Change	*r*	0.6867	1.0000	0.3534	0.0881	−0.0048	.1531	.4258	−0.2440	−0.1376
	*p*	1.9E‐07		0.0173	0.5648	0.9752	.3154	0.0035	0.1062	0.3672
LRRK2	*r*	0.2659	0.3534	1.0000	0.3008	0.2627	−0.1860	0.7456	0.0421	−0.1369
	*p*	0.0774	0.0173		0.0446	0.0812	0.2213	4.2E‐09	0.7835	0.3699
HMOX1	*r*	0.1109	0.0881	0.3008	1.0000	0.2787	−0.2744	.2109	0.2436	−0.2179
	*p*	0.4682	0.5648	0.0446		0.0638	0.0681	0.1643	0.1069	0.1505
TLR2	*r*	0.3704	−0.0048	0.2627	0.2787	1.0000	−0.3750	−0.0541	−0.2868	0.1867
	*p*	0.0123	0.9752	0.0812	0.0638		0.0111	0.7243	0.0561	0.2194
TLR8	*r*	−0.2513	0.1531	−0.1860	−.2744	−0.3750	1.0000	0.0593	−0.3250	−0.1381
	*p*	0.0958	0.3154	0.2213	0.0681	0.0111		0.6987	0.0294	0.3657
RELA	*r*	0.1847	0.4258	0.7456	0.2109	−0.0541	0.0593	1.0000	−0.0438	−.4844
	*p*	0.2245	0.0035	4.2E‐09	.1643	.7243	.6987		0.7754	0.0007
ATG7	*r*	−0.3002	−0.2440	0.0421	0.2436	−0.2868	−.3250	−0.0438	1.0000	0.2289
	*p*	0.0451	0.1062	0.7835	0.1069	0.0561	0.0294	0.7754		0.1304
GABARAPL2	*r*	−0.1110	−.1376	−0.1369	−0.2179	0.1867	−0.1381	−0.4844	0.2289	1.0000
	*p*	0.4681	0.3672	.3699	0.1505	0.2194	0.3657	0.0007	0.1304	

*r*, Pearson product moment correlation coefficient; *p*, *p* value.

**TABLE 8:** Multiple linear regression analysis of Western blot tests and MDS‐UPDRS III scores

	**Model**				**Predictors**		
**Predictors**	***r***	***r^2^***	**adj *r^2^***	***p***	***β***	**b**	***p***
LRRK2	0.3539	0.1252	0.0836	0.0602	0.3594	4.2727	0.0222
HMOX1					−0.0200	−0.2093	.8956
LRRK2	.4292	0.1842	.1453	0.0139	0.0809	0.9614	0.7010
RELA					0.3655	3.2742	0.0879
RELA	0.4329	0.1874	0.1487	0.0128	0.4692	4.2032	0.0052
GABARAPL2					0.0896	0.6156	0.5761
TLR2	0.1633	0.0267	−0.0197	0.5670	0.0613	0.2707	0.7110
TLR8					0.1761	0.7438	0.2898
TLR8	0.2562	0.0656	0.0211	0.2403	0.0825	0.3485	0.6037
ATG7					−0.2172	−2.0416	0.1757
LRRK2	0.5359	0.2872	0.1523	0.0644	0.1298	1.5436	0.6175
HMOX1					0.1797	1.8818	0.3065
TLR2					−0.2060	−0.9101	0.3216
TLR8					0.0365	0.1540	0.8350
RELA					0.3724	3.3360	0.1825
ATG7					−0.3767	−3.5403	0.0601
GABARAPL2					0.2293	1.5758	0.2410
RELA	.5007	0.2507	0.1958	0.0075	0.4919	4.4068	0.0029
ATG7					−0.2591	−2.4353	0.0701
GABARAPL2					0.1599	1.0988	0.3207
RELA	0.4819	0.2322	0.1956	0.0039	0.4159	3.7259	0.0037
ATG7					−0.2258	−2.1225	0.1026

*r*, regression coefficient; *p, p* value; *β*, standardized coefficient; b, non‐standardized coefficient.

We next assessed effects of WB intensities on total UPDRS III scores. In contrast to changes from baseline, no significant correlations with total UPDRS scores and LRRK2/HMOX1, LRRK2/RELA, and RELA/GABARAPL2 Western blot intensities (*p* = 0.2093, 0.2123, and 0.4761, respectively) (Table 9). However, two pairs of predictors, TLR2/TLR8 and TLR8/ATG7, showed significant effects on total UPDRS scores (*p* = 0.0314 and *p* = 0.0046, respectively) (Figure 5D). Western blot intensities for TLR2 and TLR8 showed significant correlation with total UPDRS scores (*r* = 0.3898, *p* = 0.0314), whereby TLR2 showed a stronger significant effect (*β* = 0.3214, *p* = 0.0421) predicting a 2.76‐point increase per unit increase in TLR2 intensity. TLR8 and ATG7 Western blot intensities correlated with total scores (*r* = 0.4757, *p* = 0.0046). Interestingly, both TLR8 and ATG7 expression were significant predictors for effects on total scores (*β* = −0.3901, *p* = 0.0095; and *β* = −0.4270, *p* = 0.0048, respectively). Moreover, assessment of all Western blot intensities as putative predictors yielded a correlation that trended to significance (*r* = 0.5335, *p* = 0.0676), and indicated that with all other predictors controlled, the strongest effect was exerted by ATG7 (*β* = −0.3606, *p* = 0.0718), and yielded a predictive decrease in total UPDRS score of 6.59 points per unit increase in ATG7 Western blot intensity.

**TABLE 9:** Multiple linear regression analysis of Western blot and MDS‐UPDRS III tests scores

	**Model**				**Predictors of the model**		
**Predictors**	***r***	***r^2^***	**adj *r^2^***	***p***	***Β***	**b**	***p***
LRRK2	.2679	0.0718	.0276	0.2093	0.2557	5.9073	0.1084
HMOX1					0.0340	0.6922	0.8284
LRRK2	0.2667	0.0711	.0269	.2123	0.2887	6.6706	0.2028
RELA					−.0306	−0.5325	0.8916
RELA	0.1863	0.0347	−0.0112	0.4761	0.1711	2.9787	.3291
GABARAPL2					−.0281	−0.3750	.8720
TLR2	0.3898	0.1519	.1115	0.0314	0.3214	2.7592	.0421
TLR8					−0.1308	−1.0742	0.3982
TLR8	0.4757	0.2263	0.1894	0.0046	−0.3901	−3.2028	0.0095
ATG7					−0.4270	−7.7984	.0048
LRRK2	0.5335	0.2846	0.1492	0.0676	0.1136	2.6248	.6626
HMOX1					0.0198	0.4034	0.9097
TLR2					0.1285	1.1034	0.5354
TLR8					−0.3034	−2.4906	0.0898
RELA					0.0901	1.5677	0.7448
ATG7					−0.3606	−6.5853	0.0718
GABARAPL2					−0.0308	−0.4114	0.8739

*r*, regression coefficient; *p*, *p* value; *β*, standardized coefficient; b, non‐standardized coefficient.

## 4 DISCUSSION

Neurodegenerative disorders that include PD, AD, ALS, Huntington's disease, and prion diseases, are an increasing global public health threat.^41^ Each is difficult to diagnose or treat, and as such, an urgent need exists to develop biomarkers that reflect both the underlying pathophysiology and responses to therapy.^42^, ^43^ These are critically important for pre‐clinical research as well as designing clinical trials. To date, reliable tests of disease progression include bioimaging with positron emission tomography (PET), single photon emission computed tomography (SPECT), magnetic resonance imaging (MRI), transcranial sonography (TCS), and magnetoencephalography (MEG).^44^ However, each of these measures have limitations such as lack of specificity, high costs, time needed, invasiveness, and lack of sensitivity to detect disease processes at the molecular level and the therapeutic effects thereof.^45^ For PD and beyond bioimaging, studies exploring genetic and biochemical biomarkers have proven helpful, but have so far failed to yield a set of definitive disease biomarkers.^46^, ^47^ The importance of *α*‐syn in PD pathogenesis directs studies to focus on measurements of total *α*‐syn, oligomeric, and modified forms of this protein in blood,^47^ cerebrospinal fluid (CSF),^48^ and in neuron‐specific extracellular vesicles.^49^ Studies have also explored *α*‐syn in various forms of PD within the gut,^50^ submandibular glands,^51^ skin,^52^ and retina^53^ for reflecting *α*‐syn as a definitive PD biomarker. While blood *α*‐syn species, neurofilament light chain, and lysosomal enzymes could supplement CSF findings, these have yet to show accuracy for following disease progression, and thus strongly supports the need for validation by other measurements.^42^, ^54^, ^55^, ^56^ Moreover, *α*‐syn has neither been consistent as a systemic biomarker for disease progression nor for response to therapy. All make reliable diagnostic and prognostic biomarkers of urgent need.

Monocytes are known to be involved in the recognition and phagocytosis of protein aggregates and debris from degenerating neurons and other cells, or from microbial organisms and their components.^57^, ^58^ In PD pathobiology, monocytes produce inflammatory cytokines in response to TLR and inflammasome stimulation by *α*‐syn or other pathogen‐ or damage‐associated molecular patterns (PAMPs and DAMPs),^58^ which, in turn, drive inflammatory responses. Monocyte‐derived cells may also play a role in the presentation of pathogenic *α*‐syn and other antigens to T lymphocytes^59^ mediated by HLA‐DR.^60^ However, PD patient monocytes are typically unchanged in number, thus pro‐inflammatory cytokine elevations result from changes of monocyte activation.^3^ Peripheral blood monocytes in PD patients present impaired functions and altered subpopulations.^61^ This implies a close link between peripheral monocytes and PD pathology. Mounting evidence confirms that peripheral inflammation triggers neuroinflammation and neurodegeneration.^62^ Therefore, both peripheral monocytes and microglia have been suggested to contribute to the inflammatory process in PD.^63^

Clinical and pathobiological diversity of PD presents major challenges in development of relevant biomarkers to monitor disease progression and disease‐modifying therapies. We have shown that GM‐CSF (sargramostim) is effective in restoring immune homeostasis and clinical improvement in PD patients, and as GM‐CSF preferentially targets myeloid lineages,^14^ we utilized monocytes‐macrophages as a means to uncover therapeutic‐related biomarkers at the molecular level. Notably, GM‐CSF not only affects macrophage immunity, but also is known to influence CNS function through altering microglia immune responses.^64^, ^65^ We posit that innate immunity via monocytes, macrophages, and microglial are linked and, in measure, orchestrate Teff and Treg cell responses. Both innate and T cell‐mediated immunity are affected during sargramostim therapy, and as such, provide protection against nigrostriatal degeneration in PD.^13^, ^14^, ^15^, ^16^ Indeed in our studies, translation to humans validated GM‐CSF activities in two Phase 1 PD clinical trials, whereby sargramostim treatment increased Treg numbers and function with improved motor function and associated brain activity.^13^, ^14^ For these reasons, we sought to characterize gene and protein activities of monocytes isolated from PD patients during immune modulatory therapy. The results showed that monocyte immune profiling could reflect operative processes in microglia, and is linked to a general adaptive immune transformative response. The results have proven to be predictive in clinical responses by monitoring UPDRS III scores during timed‐disease progression.

Herein, the monocyte transcriptome and proteome showed that sargramostim enriched different immune processes, pointing an activation of monocytes as well as a connection between innate and adaptive immune responses. Our data are in agreement with a recent study which showed enrichment of biological processes involved in regulation of immune responses in the transcriptome of human monocytes and microglia.^46^ Our findings are also consistent with previous reports depicting the activation effect of GM‐CSF on monocyte‐macrophage cell lineages.^66^ In parallel, proteomic data displayed enrichment of different cellular transport and degradative and microbicidal pathways such as endoplasmic reticulum to Golgi vesicle‐mediated transport, protein targeting to lysosome, phagosome formation, and chemokine signalling. In addition, the monocyte proteome displayed enriched cellular components related to the aforementioned biological processes, such as secretory vesicle, endocytic vesicle, phagocytic vesicle, Golgi‐associated vesicle, phagosome, and lysosome. This supports the ability of monocytes, after sargramostim treatments, to recognize danger signals (PAMPs and DAMPs), phagocytose, present antigens, and secrete both cytokines and chemokines.^58^ The interplay between innate and adaptive immunity^59^ underscores sargramostim‐based enrichment of regulation of RNA processing, splicing, and transport. All lead to the restoration of cell‐based homeostasis.

Interestingly, the sargramostim‐affected monocytic proteome was characterized by downregulation of both inflammatory and oxidative stress factors such as IL‐8, ILK, TNF, nitric oxide, and oxidative phosphorylation. Similarly, mitochondrial functions through oxidative phosphorylation^67^ also were enriched by treatment. IL‐8 is known to be an inflammatory factor involved in cell infiltration across the blood‐brain barrier (BBB)^68^ and is increased during idiopathic PD.^69^ In addition, HMOX1 promoter contains response elements which allow its induction by factors implicated in PD pathogenesis. These include IL‐1*β*, TNF‐*α*, hydrogen peroxide, 1‐methyl‐4‐phenyl‐1,2,3,6‐tetrahydropyridine (MPTP)‐like xenobiotics, and heavy metals.^70^, ^71^ Overproduction of carbon monoxide and free ferrous iron by HMOX1 supports ROS production in mitochondrial and other intracellular compartments and induces cellular injury.^72^ Moreover, prior studies suggest that modified and misfolded *α*‐syn serves as a DAMP ligand that activates myeloid phagocytes, including microglia, monocyte‐derived macrophages, and dendritic cells; by pattern recognition receptors (PRRs) such as TLR2, TLR4, and CD11b.^73^, ^74^, ^75^, ^76^ Activation of TLRs may also be induced by DAMP‐related mediators of oxidative stress such as those factors released by damaged or dying neurons.^77^, ^78^ The recognition of damage‐associated signals by PRRs plays a pivotal role, once again, in the induction of oxidative stress and inflammation in PD.^79^ Furthermore, NF‐κB activation and inflammatory signalling are chronically perturbed in disease.^80^ Activation of NF‐κB leads to the transcription of several pro‐inflammatory molecules such as IL‐1*β*, inducible nitric oxide synthase (iNOS), and TNF‐*α*.^81^, ^82^ Similarly, inhibitor of NF‐κB kinase subunit gamma (IKBKG); a subunit of the IκB kinase complex which activates NF‐κB, results in activation of inflammatory genes.^83^ All serve to exacerbate dopaminergic neurons damage in PD.^84^ Therefore, downregulation of HMOX1, TLR2, TLR8, IKBKG, and RELA proteins, as well as inhibition of several different inflammation and oxidative stress signalling pathways, support anti‐inflammatory, antioxidant, and consequently, potential neuroprotective mechanisms in PD.

The pathobiology of PD involves cyclic phases of neurodegeneration and failure of the proteasome‐ubiquitin system to clear excess *α*‐syn.^85^ Mounting evidence suggests that aggregation of *α*‐syn in PD is a consequence of impaired autophagic‐lysosomal degradation.^86^, ^87^ In turn, *α*‐syn impacts mitochondrial, lysosomal, and autophagic functions.^87^, ^88^ Subsequently, *α*‐syn misfolds, and accumulates in dopaminergic neurons preceding neuronal death. Neuronal degeneration leads to release of *α*‐syn, which is oxidatively modified, misfolded, and oligomerized, then serves to activate microglia.^89^ This shifts the brain's microenvironment and exacerbates neurodegeneration.^76^ Autophagy is an important cellular process working to counter these effects. It helps in maintaining cellular homeostasis as one of the major degradation pathways playing a pivotal role in maintaining intracellular effective protein and damaged organelle turnover.^90^ Importantly, the sargramostim‐affected monocytic proteome showed enrichment of autophagy, by activated sirtuin signalling pathway, as a possible mechanism for clearance of misfolded and aggregated proteins. Under cellular stress, including nutrient depletion or starvation, sirtuin 1 activity is increased, autophagy proteins are deacetylated, and subsequently, autophagy is induced.^91^ Autophagy related proteins (ATGs), including ATG7 and ATG8, directly interact with and are deacetylated by SIRT1.^91^ Additionally, ATG3 catalyses the formation of ATG8‐phosphatidylethanolamine (PE) conjugate which is essential for autophagy.^40^ Moreover, GABARAPs are required for autophagosomal maturation and play an important role in the degradation of autophagosomes.^92^ Our data showed upregulation of autophagy proteins ATG3, ATG7, and GABARAPL2, pointing to the restoration of normal autophagy following sargramostim treatment. On the other hand, LRRK2 (PARK8), a PD‐associated gene whose mutations account for the majority of autosomal dominant cases in PD, was found to be primarily localized in membrane microdomains, multivesicular bodies, and autophagic vesicles, and involved in different cellular signalling pathways, including autophagy.^93^ Our findings showed downregulation of LRRK2 in PD monocytes after treatment, which are in accordance with a recent study which showed increased gene expression of LRRK2 in PD monocytes compared to control monocytes and microglia.^46^

Interestingly, Western blot analysis confirmed that LRRK2, HMOX1, and TLR2 proteins were significantly downregulated in 60%–80% of patients at 2 and 6 months of sargramostim treatment, thus verifying the proteomic data. Additionally, Western blots validated downregulation of TLR8 and RELA proteins after 2 months in 60% (3/5) of patients with significant downregulation in 1/3 of patients for each protein, thus supporting the proteomic results. Moreover, Western blot analysis confirmed the downregulation of TLR8 protein after 6 months in 60% (3/5) of patients with significant downregulation in 2/3 of patients (2004 and 2005), and a near significant downregulation in the third patient (2006), which also supported the proteomic results. Interestingly, Western blot tests showed significant downregulation of RELA in 80% (4/5) of patients after 6 months. Furthermore, Western blot confirmed the upregulation of GABARAPL2 protein in 60% (3/5) of patients after 6 months. One of the major challenges of protein detection by Western blot is sensitivity to detect low‐abundance proteins in some samples. This could explain in some monocyte samples the low or normal expression of ATG7 and GABARAPL2 proteins by Western blot, while detectable by mass spectrometry, for instance, during sargramostim treatment compared to baseline. Similarly, ddPCR results confirmed that *LRRK2*, *HMOX1*, *TLR2*, *TLR8* genes were significantly downregulated in 60%–100% of patients at 2 and 6 months of sargramostim treatment, and supported both proteomic and Western blot analyses. In addition, ddPCR validated that *RELA* gene was significantly downregulated in 60% of patients after 2 months, corroborating the relative protein levels as determined by proteomic and Western blot results. *RELA* gene expression was not altered notably after 6 months, thus supporting the proteomic results for RELA protein. Interestingly, ddPCR confirmed that *ATG7* gene expression was significantly upregulated in 80% of patients after 6 months, supporting the proteomic data. In accordance with 6 month‐Western blot results, ddPCR also confirmed upregulation of *GABARAPL2* gene in 60% of patients which supported the proteomic analysis.

We posit that the discrepancy between mRNA and protein expression of some genes, at 2 or 6 months compared to baseline, may be due to the fact that RNA and protein represent different steps of the multi‐stepped cellular genetic information flow process, in which they are dynamically produced and degraded.^94^ In this study, monocytes were collected at baseline and at 2 and 6 months of treatment, thus, mRNA and protein expression were static, not dynamic, which reflects the expression level at the moment of sample collection. The spatial and temporal variations of mRNAs, as well as the local availability of resources for protein synthesis, strongly affect the relationship between protein levels and their coding transcripts.^95^ Overall, our data suggests the potential for using gene and/or protein expression levels of LRRK2, HMOX1, TLR2, TLR8, RELA, ATG7, and GABARAPL2 to follow therapeutic response with sargramostim during progression of PD. Therefore, these genes/proteins may serve as potential biomarkers to predict therapeutic response in PD patients treated with sargramostim or similar immunomodulatory therapies. Significantly, our data showed the predictive potential of LRRK2 gene and protein expression for UPDRS III scores and changes in scores. Similarly, *HMOX1*, *TLR2*, and *ATG7* gene expression, as well as RELA, TLR2, and ATG7 protein expression, showed predictive potential for UPDRS III scores and changes in scores, thus suggesting their utility as putative biomarkers for sargramostim therapy. A limitation of the current study is the small sample size employed which may overestimate the veracity of the conclusions. Due to the limited study participants, the recorded difference in the gene/protein expression of the examined putative biomarkers will require larger sample analysis in order to confirm the results recorded in the current evaluation.

To our knowledge, this study is the first to address the association between monocyte profiles and both clinical motor function and disease progression during immune modulatory therapy. We demonstrated that the monocyte signature profile would present relevant biomarkers to monitor the clinical improvement and disease progression during anti‐parkinsonism immune modulatory therapy trials. Despite the small sample size of patients and lack of placebo controls, the utilization of patients’ baseline samples before starting the treatment allowed for before and after treatment comparisons and temporal evaluations. In addition, the nature of the study as an unblinded, open‐label phase 1 represents a limitation of this study because it introduces a significant risk for bias. Additionally, all enrolled study participants were male. However, these limitations require further validation in a larger double blinded placebo‐controlled phase II trial that includes both sexes.

## CONFLICT OF INTEREST

The authors declare that there is no conflict of interest that could be perceived as prejudicing the impartiality of the research reported.

## Supporting information

Supporting Information

Supporting Information

Supporting Information

Supporting Information

Supporting Information

Supporting Information

Supporting Information

Supporting Information

Supporting Information

Supporting Information

Supporting Information

Supporting Information

Supporting Information

Supporting Information

Supporting Information

## Data Availability

All data associated with this study are present in the manuscript or the supplementary information. Additional files are available in figshare at https://doi.org/10.6084/m9.figshare.19380230.

## References

[1] Jain S . Multi‐organ autonomic dysfunction in Parkinson disease. Parkinsonism Relat Disord. 2011;17(2):77‐83. 20851033 10.1016/j.parkreldis.2010.08.022 PMC3021587

[2] Houser MC , Tansey MG . The gut‐brain axis: is intestinal inflammation a silent driver of Parkinson's disease pathogenesis? NPJ Parkinsons Dis. 2017 **;** 3:3. 28649603 10.1038/s41531-016-0002-0 PMC5445611

[3] Nissen SK , Shrivastava K , Schulte C , et al. Alterations in blood monocyte functions in Parkinson's disease. Mov Disord 2019;34(11):1711‐1721. 31449711 10.1002/mds.27815

[4] Brochard V , Combadiere B , Prigent A , et al. Infiltration of CD4+ lymphocytes into the brain contributes to neurodegeneration in a mouse model of Parkinson disease. J Clin Invest. 2009;119(1):182‐192. 19104149 10.1172/JCI36470 PMC2613467

[5] Rocha EM , De Miranda B , Sanders LH . Alpha‐synuclein: pathology, mitochondrial dysfunction and neuroinflammation in Parkinson's disease. Neurobiol Dis. 2018;109(Pt B):249‐257. 28400134 10.1016/j.nbd.2017.04.004

[6] Jankovic J , Tan EK . Parkinson's disease: etiopathogenesis and treatment. J Neurol Neurosurg Psychiatry. 2020;91(8):795‐808. 32576618 10.1136/jnnp-2019-322338

[7] Harms AS , Thome AD , Yan Z , et al. Peripheral monocyte entry is required for alpha‐synuclein induced inflammation and neurodegeneration in a model of Parkinson disease. Exp Neurol. 2018;300:179‐187. 29155051 10.1016/j.expneurol.2017.11.010 PMC5759972

[8] Waschbisch A , Schroder S , Schraudner D , et al. Pivotal role for CD16+ monocytes in immune surveillance of the central nervous system. J Immunol. 2016;196(4):1558‐1567. 26746191 10.4049/jimmunol.1501960

[9] Thome AD , Atassi F , Wang J , et al. Ex vivo expansion of dysfunctional regulatory T lymphocytes restores suppressive function in Parkinson's disease. NPJ Parkinsons Dis. 2021;7(1):41. 33986285 10.1038/s41531-021-00188-5 PMC8119976

[10] Machhi J , Kevadiya BD , Muhammad IK , et al. Harnessing regulatory T cell neuroprotective activities for treatment of neurodegenerative disorders. Mol Neurodegener. 2020;15(1):32. 32503641 10.1186/s13024-020-00375-7 PMC7275301

[11] Schabitz WR , Kruger C , Pitzer C , et al. A neuroprotective function for the hematopoietic protein granulocyte‐macrophage colony stimulating factor (GM‐CSF). J Cereb Blood Flow Metab. 2008;28(1):29‐43. 17457367 10.1038/sj.jcbfm.9600496

[12] Schutt CR , Gendelman HE , Mosley RL . Tolerogenic bone marrow‐derived dendritic cells induce neuroprotective regulatory T cells in a model of Parkinson's disease. Mol Neurodegener. 2018;13(1):26. 29783988 10.1186/s13024-018-0255-7 PMC5963189

[13] Gendelman HE , Zhang Y , Santamaria P , et al. Evaluation of the safety and immunomodulatory effects of sargramostim in a randomized, double‐blind phase 1 clinical Parkinson's disease trial. NPJ Parkinsons Dis. 2017;3:10. 28649610 10.1038/s41531-017-0013-5 PMC5445595

[14] Olson KE , Namminga KL , Lu Y , et al. Safety, tolerability, and immune‐biomarker profiling for year‐long sargramostim treatment of Parkinson's disease. EBioMedicine. 2021;67:103380. 34000620 10.1016/j.ebiom.2021.103380 PMC8138485

[15] Kosloski LM , Kosmacek EA , Olson KE , Mosley RL , Gendelman HE . GM‐CSF induces neuroprotective and anti‐inflammatory responses in 1‐methyl‐4‐phenyl‐1,2,3,6‐tetrahydropyridine intoxicated mice. J Neuroimmunol 2013;265(1‐2):1‐10. 24210793 10.1016/j.jneuroim.2013.10.009 PMC3872482

[16] Olson KE , Namminga KL , Schwab AD , et al. Neuroprotective activities of long‐acting granulocyte‐macrophage colony‐stimulating factor (mPDM608) in 1‐methyl‐4‐phenyl‐1,2,3,6‐tetrahydropyridine‐intoxicated mice. Neurotherapeutics. 2020;17(4):1861‐1877. 32638217 10.1007/s13311-020-00877-8 PMC7851309

[17] Mosley RL , Lu Y , Olson KE , et al. A synthetic agonist to vasoactive intestinal peptide receptor‐2 induces regulatory T cell neuroprotective activities in models of Parkinson's disease. Front Cell Neurosci. 2019;13:421. 31619964 10.3389/fncel.2019.00421 PMC6759633

[18] Zheng SG , Wang JH , Koss MN , Quismorio F, Jr. , Gray JD , Horwitz DA . CD4+ and CD8+ regulatory T cells generated ex vivo with IL‐2 and TGF‐beta suppress a stimulatory graft‐versus‐host disease with a lupus‐like syndrome. J Immunol. 2004;172(3):1531‐1539. 14734731 10.4049/jimmunol.172.3.1531

[19] Cook AD , Louis C , Robinson MJ , Saleh R , Sleeman MA , Hamilton JA . Granulocyte macrophage colony‐stimulating factor receptor alpha expression and its targeting in antigen‐induced arthritis and inflammation. Arthritis Res Ther. 2016;18(1):287. 27908288 10.1186/s13075-016-1185-9 PMC5134062

[20] Hamilton JA , Cook AD , Tak PP . Anti‐colony‐stimulating factor therapies for inflammatory and autoimmune diseases. Nat Rev Drug Discov. 2016;16(1):53‐70. 28031576 10.1038/nrd.2016.231

[21] Potter H , Woodcock JH , Boyd TD , et al. Safety and efficacy of sargramostim (GM‐CSF) in the treatment of Alzheimer's disease. Alzheimers Dement (N Y) 2021;7(1):e12158. 33778150 10.1002/trc2.12158 PMC7988877

[22] Lotankar S , Prabhavalkar KS , Bhatt LK . Biomarkers for Parkinson's disease: recent advancement. Neurosci Bull. 2017;33(5):585‐597. 28936761 10.1007/s12264-017-0183-5 PMC5636742

[23] Arainga M , Guo D , Wiederin J , Ciborowski P , McMillan J , Gendelman HE . Opposing regulation of endolysosomal pathways by long‐acting nanoformulated antiretroviral therapy and HIV‐1 in human macrophages. Retrovirology 2015;12:5. 25608975 10.1186/s12977-014-0133-5 PMC4307176

[24] Gao L , Kumar V , Vellichirammal NN , et al. Functional, proteomic and bioinformatic analyses of Nrf2‐ and Keap1‐ null skeletal muscle. J Physiol. 2020;598(23):5427‐5451. 32893883 10.1113/JP280176 PMC7749628

[25] Benjamini Y , Drai D , Elmer G , Kafkafi N , Golani I . Controlling the false discovery rate in behavior genetics research. Behav Brain Res. 2001;125(1‐2):279‐284. 11682119 10.1016/s0166-4328(01)00297-2

[26] Bindea G , Mlecnik B , Hackl H , et al. ClueGO: a Cytoscape plug‐in to decipher functionally grouped gene ontology and pathway annotation networks. Bioinformatics 2009;25(8):1091‐1093. 19237447 10.1093/bioinformatics/btp101 PMC2666812

[27] Gaurav R , Mikuls TR , Thiele GM , et al. High‐throughput analysis of lung immune cells in a combined murine model of agriculture dust‐triggered airway inflammation with rheumatoid arthritis. PLoS One 2021;16(2):e0240707. 33577605 10.1371/journal.pone.0240707 PMC7880471

[28] Benjamini Y , Krieger AM , Yekutieli D . Adaptive linear step‐up procedures that control the false discovery rate. Biometrika 2006;93(3):491‐507.

[29] Xu E , Boddu R , Abdelmotilib HA , et al. Pathological alpha‐synuclein recruits LRRK2 expressing pro‐inflammatory monocytes to the brain. Mol Neurodegener. 2022;17(1):7. 35012605 10.1186/s13024-021-00509-5 PMC8751347

[30] Grozdanov V , Bousset L , Hoffmeister M , et al. Increased immune activation by pathologic alpha‐synuclein in Parkinson's disease. Ann Neurol 2019;86(4):593‐606. 31343083 10.1002/ana.25557

[31] Lee IH . Mechanisms and disease implications of sirtuin‐mediated autophagic regulation. Exp Mol Med. 2019;51(9):1‐11. 10.1038/s12276-019-0302-7 PMC6802627 31492861

[32] Clarke CJ , Hales A , Hunt A , Foxwell BM . IL‐10‐mediated suppression of TNF‐alpha production is independent of its ability to inhibit NF kappa B activity. Eur J Immunol. 1998;28(5):1719‐1726. 9603479 10.1002/(SICI)1521-4141(199805)28:05<1719::AID-IMMU1719>3.0.CO;2-Q

[33] Dickensheets HL , Donnelly RP . IFN‐gamma and IL‐10 inhibit induction of IL‐1 receptor type I and type II gene expression by IL‐4 and IL‐13 in human monocytes. J Immunol. 1997;159(12):6226‐33. 9550426

[34] Nibbs RJ , Graham GJ . Immune regulation by atypical chemokine receptors. Nat Rev Immunol. 2013;13(11):815‐829. 24319779 10.1038/nri3544

[35] Li RY , Guan J , Zhou S . Boosting scRNA‐seq data clustering by cluster‐aware feature weighting. BMC Bioinformatics. 2021;22(suppl 6):130. 34078287 10.1186/s12859-021-04033-7 PMC8171019

[36] Beyer M , Mallmann MR , Xue J , et al. High‐resolution transcriptome of human macrophages. PLoS One 2012;7(9):e45466. 23029029 10.1371/journal.pone.0045466 PMC3448669

[37] Alonso MN , Gregorio JG , Davidson MG , Gonzalez JC , Engleman EG . Depletion of inflammatory dendritic cells with anti‐CD209 conjugated to saporin toxin. Immunol Res. 2014;58(2‐3):374‐377. 24781193 10.1007/s12026-014-8511-6 PMC4160227

[38] Xiong J . Atg7 in development and disease: panacea or Pandora's box? Protein Cell. 2015;6(10):722‐734. 26404030 10.1007/s13238-015-0195-8 PMC4598325

[39] Szalai P , Hagen LK , Saetre F , et al. Autophagic bulk sequestration of cytosolic cargo is independent of LC3, but requires GABARAPs. Exp Cell Res. 2015;333(1):21‐38. 25684710 10.1016/j.yexcr.2015.02.003

[40] Yamada Y , Suzuki NN , Hanada T , et al. The crystal structure of Atg3, an autophagy‐related ubiquitin carrier protein (E2) enzyme that mediates Atg8 lipidation. J Biol Chem. 2007;282(11):8036‐8043. 17227760 10.1074/jbc.M611473200

[41] Collaborators, G. B. D. N. Global, regional, and national burden of neurological disorders, 1990–2016: a systematic analysis for the Global Burden of Disease Study 2016. Lancet Neurol. 2019;18(5):459‐480. 30879893 10.1016/S1474-4422(18)30499-X PMC6459001

[42] Ganguly U , Singh S , Pal S , et al. Alpha‐synuclein as a biomarker of Parkinson's disease: good, but not good enough. Front Aging Neurosci. 2021;13:702639. 34305577 10.3389/fnagi.2021.702639 PMC8298029

[43] Verber NS , Shepheard SR , Sassani M , et al. Biomarkers in motor neuron disease: a state of the art review. Front Neurol. 2019;10:291. 31001186 10.3389/fneur.2019.00291 PMC6456669

[44] Niethammer M , Feigin A , Eidelberg D . Functional neuroimaging in Parkinson's disease. Cold Spring Harb Perspect Med. 2012;2(5):a009274. 22553499 10.1101/cshperspect.a009274 PMC3331691

[45] Rahmim A , Zaidi H . PET versus SPECT: strengths, limitations and challenges. Nucl Med Commun. 2008;29(3):193‐207. 18349789 10.1097/MNM.0b013e3282f3a515

[46] Schlachetzki JCM , Prots I , Tao J , et al. Author correction: a monocyte gene expression signature in the early clinical course of Parkinson's disease. Sci Rep. 2020;10(1):6261. 32253373 10.1038/s41598-020-62928-6 PMC7136204

[47] Gao L , Tang H , Nie K , et al. Cerebrospinal fluid alpha‐synuclein as a biomarker for Parkinson's disease diagnosis: a systematic review and meta‐analysis. Int J Neurosci. 2015;125(9):645‐654. 25202803 10.3109/00207454.2014.961454

[48] van Dijk KD , Bidinosti M , Weiss A , Raijmakers P , Berendse HW , van de Berg WD . Reduced alpha‐synuclein levels in cerebrospinal fluid in Parkinson's disease are unrelated to clinical and imaging measures of disease severity. Eur J Neurol. 2014;21(3):388‐394. 23631635 10.1111/ene.12176

[49] Gustafsson G , Loov C , Persson E , et al. Secretion and uptake of alpha‐synuclein via extracellular vesicles in cultured cells. Cell Mol Neurobiol 2018;38(8):1539‐1550. 30288631 10.1007/s10571-018-0622-5 PMC6223723

[50] Schaeffer E , Kluge A , Bottner M , et al. Alpha synuclein connects the gut‐brain axis in Parkinson's disease patients ‐ a view on clinical aspects, cellular pathology and analytical methodology. Front Cell Dev Biol. 2020;8:573696. 33015066 10.3389/fcell.2020.573696 PMC7509446

[51] Campo F , Carletti R , Fusconi M , et al. Alpha‐synuclein in salivary gland as biomarker for Parkinson's disease. Rev Neurosci 2019;30(5):455‐462. 30471223 10.1515/revneuro-2018-0064

[52] Kim JY , Illigens BM , McCormick MP , Wang N , Gibbons CH . Alpha‐synuclein in skin nerve fibers as a biomarker for alpha‐synucleinopathies. J Clin Neurol. 2019;15(2):135‐142. 30938106 10.3988/jcn.2019.15.2.135 PMC6444158

[53] Ortuno‐Lizaran I , Beach TG , Serrano GE , Walker DG , Adler CH , Cuenca N . Phosphorylated alpha‐synuclein in the retina is a biomarker of Parkinson's disease pathology severity. Mov Disord. 2018;33(8):1315‐1324. 29737566 10.1002/mds.27392 PMC6146055

[54] Gaetani L , Blennow K , Calabresi P , Di Filippo M , Parnetti L , Zetterberg H . Neurofilament light chain as a biomarker in neurological disorders. J Neurol Neurosurg Psychiatry. 2019;90(8):870‐881. 30967444 10.1136/jnnp-2018-320106

[55] Parnetti L , Gaetani L , Eusebi P , et al. CSF and blood biomarkers for Parkinson's disease. Lancet Neurol. 2019;18(6):573‐586. 30981640 10.1016/S1474-4422(19)30024-9

[56] Xicoy H , Penuelas N , Vila M , Laguna A . Autophagic‐ and lysosomal‐related biomarkers for Parkinson's disease: lights and shadows. Cells 2019;8(11):1317. 10.3390/cells8111317 PMC6912814 31731485

[57] Bliederhaeuser C , Grozdanov V , Speidel A , et al. Age‐dependent defects of alpha‐synuclein oligomer uptake in microglia and monocytes. Acta Neuropathol. 2016;131(3):379‐391. 26576561 10.1007/s00401-015-1504-2

[58] Codolo G , Plotegher N , Pozzobon T , et al. Triggering of inflammasome by aggregated alpha‐synuclein, an inflammatory response in synucleinopathies. PLoS One. 2013;8(1):e55375. 23383169 10.1371/journal.pone.0055375 PMC3561263

[59] Sulzer D , Alcalay RN , Garretti F , et al. T cells from patients with Parkinson's disease recognize alpha‐synuclein peptides. Nature 2017;546(7660):656‐661. 28636593 10.1038/nature22815 PMC5626019

[60] Kannarkat GT , Cook DA , Lee JK , et al. Common genetic variant association with altered HLA expression, synergy with pyrethroid exposure, and risk for Parkinson's disease: an observational and case‐control study. NPJ Parkinsons Dis. 2015;1:15002. 27148593 10.1038/npjparkd.2015.2 PMC4853162

[61] da Silva DJ , Borges AF , Souza PO , et al. Decreased toll‐like receptor 2 and toll‐like receptor 7/8‐induced cytokines in Parkinson's disease patients. Neuroimmunomodulation 2016;23(1):58‐66. 26886382 10.1159/000443238

[62] Qin XY , Zhang SP , Cao C , Loh YP , Cheng Y . Aberrations in peripheral inflammatory cytokine levels in Parkinson disease: a systematic review and meta‐analysis. JAMA Neurol. 2016;73(11):1316‐1324. 27668667 10.1001/jamaneurol.2016.2742

[63] Tansey MG , Romero‐Ramos M . Immune system responses in Parkinson's disease: early and dynamic. Eur J Neurosci. 2019;49(3):364‐383. 30474172 10.1111/ejn.14290 PMC6391192

[64] Easley‐Neal C , Foreman O , Sharma N , Zarrin AA , Weimer RM . CSF1R ligands IL‐34 and CSF1 are differentially required for microglia development and maintenance in white and gray matter brain regions. Front Immunol. 2019;10:2199. 31616414 10.3389/fimmu.2019.02199 PMC6764286

[65] Esen N , Kielian T . Effects of low dose GM‐CSF on microglial inflammatory profiles to diverse pathogen‐associated molecular patterns (PAMPs). J Neuroinflammation. 2007;4:10. 17374157 10.1186/1742-2094-4-10 PMC1839084

[66] Ushach I , Zlotnik A . Biological role of granulocyte macrophage colony‐stimulating factor (GM‐CSF) and macrophage colony‐stimulating factor (M‐CSF) on cells of the myeloid lineage. J Leukoc Biol. 2016;100(3):481‐489. 27354413 10.1189/jlb.3RU0316-144R PMC4982611

[67] Bergman O , Ben‐Shachar D . Mitochondrial oxidative phosphorylation system (OXPHOS) deficits in schizophrenia: possible interactions with cellular processes. Can J Psychiatry. 2016;61(8):457‐469. 27412728 10.1177/0706743716648290 PMC4959648

[68] Hammond ME , Lapointe GR , Feucht PH , et al. IL‐8 induces neutrophil chemotaxis predominantly via type I IL‐8 receptors. J Immunol. 1995;155(3):1428‐1433. 7636208

[69] Yilmaz R , Strafella AP , Bernard A , et al. Serum inflammatory profile for the discrimination of clinical subtypes in Parkinson's disease. Front Neurol. 2018;9:1123. 30622507 10.3389/fneur.2018.01123 PMC6308160

[70] Schipper HM , Song W . A heme oxygenase‐1 transducer model of degenerative and developmental brain disorders. Int J Mol Sci. 2015;16(3):5400‐5419. 25761244 10.3390/ijms16035400 PMC4394483

[71] Schipper HM , Song W , Tavitian A , Cressatti M . The sinister face of heme oxygenase‐1 in brain aging and disease. Prog Neurobiol. 2019;172:40‐70. 30009872 10.1016/j.pneurobio.2018.06.008

[72] Schipper HM . Brain iron deposition and the free radical‐mitochondrial theory of ageing. Ageing Res Rev. 2004;3(3):265‐301. 15231237 10.1016/j.arr.2004.02.001

[73] Hou L , Bao X , Zang C , et al. Integrin CD11b mediates alpha‐synuclein‐induced activation of NADPH oxidase through a Rho‐dependent pathway. Redox Biol. 2018;14:600‐608. 29154191 10.1016/j.redox.2017.11.010 PMC5975218

[74] Kim C , Ho DH , Suk JE , et al. Neuron‐released oligomeric alpha‐synuclein is an endogenous agonist of TLR2 for paracrine activation of microglia. Nat Commun. 2013;4:1562. 23463005 10.1038/ncomms2534 PMC4089961

[75] Fellner L , Irschick R , Schanda K , et al. Toll‐like receptor 4 is required for alpha‐synuclein dependent activation of microglia and astroglia. Glia. 2013;61(3):349‐360. 23108585 10.1002/glia.22437 PMC3568908

[76] Schwab AD , Thurston MJ , Machhi J , et al. Immunotherapy for Parkinson's disease. Neurobiol Dis. 2020;137:104760. 31978602 10.1016/j.nbd.2020.104760 PMC7933730

[77] Braza F , Brouard S , Chadban S , Goldstein DR . Role of TLRs and DAMPs in allograft inflammation and transplant outcomes. Nat Rev Nephrol. 2016;12(5):281‐290. 27026348 10.1038/nrneph.2016.41 PMC7952035

[78] Campolo M , Filippone A , Biondo C , et al. TLR7/8 in the pathogenesis of Parkinson's disease. Int J Mol Sci. 2020;21(24):9384. 10.3390/ijms21249384 PMC7763162 33317145

[79] Roh JS , Sohn DH . Damage‐associated molecular patterns in inflammatory diseases. Immune Netw. 2018;18(4):e27. 30181915 10.4110/in.2018.18.e27 PMC6117512

[80] Ahmed AU , Sarvestani ST , Gantier MP , Williams BR , Hannigan GE . Integrin‐linked kinase modulates lipopolysaccharide‐ and Helicobacter pylori‐induced nuclear factor kappaB‐activated tumor necrosis factor‐alpha production via regulation of p65 serine 536 phosphorylation. J Biol Chem. 2014;289(40):27776‐27793. 25100717 10.1074/jbc.M114.574541 PMC4183813

[81] Dasgupta S , Jana M , Liu X , Pahan K . Role of very‐late antigen‐4 (VLA‐4) in myelin basic protein‐primed T cell contact‐induced expression of proinflammatory cytokines in microglial cells. J Biol Chem. 2003;278(25):22424‐22431. 12690109 10.1074/jbc.M301789200 PMC1955481

[82] Liu X , Jana M , Dasgupta S , et al. Human immunodeficiency virus type 1 (HIV‐1) tat induces nitric‐oxide synthase in human astroglia. J Biol Chem. 2002;277(42):39312‐39319. 12167619 10.1074/jbc.M205107200 PMC2041896

[83] Rothwarf DM , Zandi E , Natoli G , Karin M . IKK‐gamma is an essential regulatory subunit of the IkappaB kinase complex. Nature 1998;395(6699):297‐300. 9751060 10.1038/26261

[84] Kirkley KS , Popichak KA , Hammond SL , Davies C , Hunt L , Tjalkens RB . Genetic suppression of IKK2/NF‐kappaB in astrocytes inhibits neuroinflammation and reduces neuronal loss in the MPTP‐probenecid model of Parkinson's disease. Neurobiol Dis. 2019;127:193‐209. 30818064 10.1016/j.nbd.2019.02.020 PMC6588478

[85] Lim KL , Tan JM . Role of the ubiquitin proteasome system in Parkinson's disease. BMC Biochem. 2007;8(suppl 1):S13. 18047737 10.1186/1471-2091-8-S1-S13 PMC2106364

[86] Hou X , Watzlawik JO , Fiesel FC , Springer W . Autophagy in Parkinson's disease. J Mol Biol. 2020;432(8):2651‐2672. 32061929 10.1016/j.jmb.2020.01.037 PMC7211126

[87] Xilouri M , Brekk OR , Stefanis L . Autophagy and alpha‐synuclein: relevance to Parkinson's disease and related synucleopathies. Mov Disord. 2016;31(2):178‐192. 26813776 10.1002/mds.26477

[88] Ryan BJ , Hoek S , Fon EA , Wade‐Martins R . Mitochondrial dysfunction and mitophagy in Parkinson's: from familial to sporadic disease. Trends Biochem Sci 2015;40(4):200‐210. 25757399 10.1016/j.tibs.2015.02.003

[89] Thomas MP , Chartrand K , Reynolds A , Vitvitsky V , Banerjee R , Gendelman HE . Ion channel blockade attenuates aggregated alpha synuclein induction of microglial reactive oxygen species: relevance for the pathogenesis of Parkinson's disease. J Neurochem. 2007;100(2):503‐519. 17241161 10.1111/j.1471-4159.2006.04315.x

[90] Kocaturk NM , Gozuacik D . Crosstalk between mammalian autophagy and the ubiquitin‐proteasome system. Front Cell Dev Biol. 2018;6:128. 30333975 10.3389/fcell.2018.00128 PMC6175981

[91] Lee IH , Cao L , Mostoslavsky R , et al. A role for the NAD‐dependent deacetylase Sirt1 in the regulation of autophagy. Proc Natl Acad Sci U S A. 2008;105(9):3374‐3379. 18296641 10.1073/pnas.0712145105 PMC2265142

[92] Sasai M , Sakaguchi N , Ma JS , et al. Essential role for GABARAP autophagy proteins in interferon‐inducible GTPase‐mediated host defense. Nat Immunol. 2017;18(8):899‐910. 28604719 10.1038/ni.3767

[93] Rideout HJ , Stefanis L . The neurobiology of LRRK2 and its role in the pathogenesis of Parkinson's disease. Neurochem Res. 2014;39(3):576‐592. 23729298 10.1007/s11064-013-1073-5

[94] Wang D . Discrepancy between mRNA and protein abundance: insight from information retrieval process in computers. Comput Biol Chem. 2008;32(6):462‐468. 18757239 10.1016/j.compbiolchem.2008.07.014 PMC2637108

[95] Liu Y , Beyer A , Aebersold R . On the dependency of cellular protein levels on mRNA abundance. Cell 2016;165(3):535‐550. 27104977 10.1016/j.cell.2016.03.014

