# Supplementary material for: Monocyte biomarkers define sargramostim treatment outcomes for Parkinson's disease
Source: Clin Transl Med. 2022 Jul 8;12(7):e958. doi: 10.1002/ctm2.958 (PMC9270000; doi:10.1002/ctm2.958)
Supplement: Supplementary file 1 — Supporting Information [file CTM2-12-e958-s012.docx]

Supplementary information

**Monocyte biomarkers define sargramostim treatment outcomes for Parkinson’s disease**

Mai M. Abdelmoaty, Jatin Machhi, Pravin Yeapuri, Farah Shahjin, Vikas Kumar, Katherine E. Olson, R. Lee Mosley^*^ and Howard E. Gendelman^**^

**Corresponding authors:** ^*^R. Lee Mosley (for correspondence), Department of Pharmacology and Experimental Neuroscience, University of Nebraska Medical Center, Omaha, NE 68198-5800; phone: 402-559-2510; fax: 402-559-3744; email: [rlmosley@unmc.edu](mailto:rlmosley@unmc.edu). **^**^**Howard E. Gendelman (for submission and correspondence), Department of Pharmacology and Experimental Neuroscience, University of Nebraska Medical Center, Omaha, NE 68198-5800; phone: 402-559-8920; fax: 402-559-3744; email: [hegendel@unmc.edu](mailto:hegendel@unmc.edu)

**This file includes:**

Materials and Methods

Figs. S1 to S5

Legends for Additional files 1 to 14

**Materials and Methods**

**Monocyte centrifugal elutriation**

**1] Preparation for elutriation**

1. Assemble chambers and 3 transfer tubes. Follow diagram, starting at the bottom. Lubricate O-rings using Beckman vacuum grease silicon.
2. Ready the centrifuge. Turn on. The settings should remain from previous time. Time = hold (H); Temp = 18o – 25 oC; RPM = 1960. Attach chambers to rotor and secure cable to rotor. Connecting tubing: Out = top; In: side.
3. Clean system tubing: Replace stopcock and syringe. Place new 1 ml aspirating pipette in a bottle of 95% EtOH. Make sure pipette is all the way down to the bottom of bottle but not touching it. Replace new short 1 ml pipette at the outlet. Turn on pump (forward) and flush with EtOH with speed setting @ 2. You should see flow into waste collection. Invert chamber, tap to free any bubbles.
4. After ~200 ml of EtOH has run through the system, turn off the pump. Replace EtOH with 1L bottle of PBS (always keep this bottle filled). Turn on the pump (set @ 2). Pour PBS into the syringe and elute this to rid any air bubbles that might be in the stopcock. Close the stopcock before all of the PBS has drained and continue to run PBS through the system. Make sure you have enough PBS on hand.
5. Continue the PBS flush for ~400 ml (speed setting @2). During this flush, run centrifuge at 1960 rpm, 18 oC (recall program #1), pinching tubing continuously to eliminate any trapped air bubbles in the tubing. When the speed gets up to 1960 rpm, stop the centrifuge, and turn the pump up to 4.0 to make sure it can handle the pressure. Turn the pump back down to 1.0.
6. Check chamber for any trapped air bubble. Repeat step 5 to rid of air bubbles. Turn off the pump until ready for elutriation. (Pump should always be on before and after centrifuge operation).
7. Turn on Coulter counter. Empty waste if more than half full and fill Isoton chamber 2/3 full.
8. Flush electrode chamber with Isoton in cup (10 ml). Press START to count. Count should be ~500.
9. To count cells, add 10 ml Isoton to 20 µl cells. Place under electrode. Press START to count. Press OUTPUT to read count number. Cell concentration (cells/ml) = raw count x 103 x 10 (dilution factor).

**2] Elutriation**

**A. Preparation of mononuclear cells**

1. Aliquot 10 ml of LSM (Lymphocyte Separation Medium) into each of 10 50ml centrifuge tubes. Do not get LSM on the sides of the tubes. Cover until ready to use.
2. When leukopack arrives, massage pack to mix anticoagulant. Save packing sheet. Alcohol wash & air-dry scissors and the long tube at the top of the pack. Snip with scissors, and gently decant into a 250 ml centrifuge tube. Bring the volume up to 250 ml with PBS. Pipet to mix gently.
3. Layer 25 ml blood carefully onto 10 ml LSM.
4. Spin at 3000 rpm, 20 min., 18 oC, no brake.
5. Aspirate the plasma to 20 ml.

6. Collect the white cell layer into a 250 ml conical tube.

7. Resuspend the cells well, then add PBS to neck of tube.

8. Wash the cells by spinning at 1200 rpm, 10 min, 18oC.

9. Aspirate the supernatant to 50 ml (instead of aspirating, you can also transfer the supernatant to a new tube and spin down again.)

10. Repeat step 7 to 8

11. Aspirate the supernatant to 25 ml.

12. Resuspend cells well. Make a 1:10 dilution (10 µl cells + 90 µl PBS) and take PBMC count* and volume.

13. Put cells in syringe.

**B. Separating Cells**

1. Turn pump on with speed setting @ 0.9. Make sure PBS bottle is full.

2. Turn on centrifuge.

3. When the rotor has reached 1960 rpm, load PBMCs (close tubing to PBS)

4. Once loaded, wait for 10-15 min. to allow cells to separate by weight (observe cells in chamber, if overflow turn pump down half a point. Watch and turn back up when it settles down).

5. Turn pump to 1.0, wait for 2 min. to rid of red blood cells.

6. Turn pump to 1.1, collect one 250 ml conical tube PBL.

7. Turn pump to 1.2, and collect another.

8. Take counts. When counter show less than 500 then increase pump 0.05 at a time until all PBLs are off.

9. When a profile of monocytes starts to show, put the tube to collect MO in, stop the centrifuge, turn the dial to 4.0.

10. Centrifuge cells down at 1200 rpm, 10 min., 18 oC.

11. Aspirate supernatant to 25 ml. Resuspend cells well. Make a 1:10 dilution. PBLs: 10 µl cells + 90 µl PBS MO: 50 µl + 450 µl PBS.

12. Take cells count and volume.

13. Prepare 2 cytospins for MO: 200µl PBS + 50 µl MO and 200 µl PBS + 100 µl MO.

**C. Cell counting**

Add 10 ml Isoton to 20 µl cells. Place under electrode. Press START to count. Press OUTPUT to see cell count and profile.

**3] Cleaning after elutriation**

**A. Centrifuge**

1. Run 400 ml PBS through the system with pump set at 2.

2. Run 200 ml EtOH afterward.

3. Disassemble the chamber, clean the chamber by first soak in 10% Solution 555 for 10 min. then rinse off with water and EtOH at the end.

4. Clean all others with EtOH.

5. Turn off centrifuge.

6. Clean hood with EtOH.

7. Empty vacuum flask, make sure it was bleached before going down the sink. Rinse and fill with bleach and assemble.

**B. Coulter Counter**

1. Count 2x with Coulter Clenz. Leave cup in there.

2. Turn all three instruments off.

3. Wipe surface off with EtOH.

**Single-cell RNA-sequencing data processing**

Basecall (BCL) files were generated through 10xGenomics Chromium Single cell 3’ Solution followed by RNA Sequencing using Nextseq 500 and Nextseq 550 as previously described.^1^ Cellranger mkfastq was used for demultiplexing and to convert BCL files into FASTQ files. FASTQ files were run through Cellranger count to perform alignment (using STAR aligner), filtering, and unique molecular identifier (UMI) counting. Chromium cellular barcodes were used to generate gene-barcode matrices, perform clustering, and do gene expression analyses. Cellranger aggr was used to normalize and pool the results from different samples, followed by the application of Principal Components Analysis (PCA) to change the dimensionality of the data sets. t-SNE (t-Stochastic Neighbor Embedding) was used to visualize the data in a 2-D space. The tests generate p values which were adjusted for multiple testing using the BH procedure to control the FDR. A variant of the negative binomial exact test was performed for the differential expression based on the [published sSeq](https://academic.oup.com/bioinformatics/article/29/10/1275/259212/Shrinkage-estimation-of-dispersion-in-Negative) method (<https://kb.10xgenomics.com/hc/en-us/articles/115003936051-Can-you-give-more-information-on-the-statistical-test-used-for-differential-expression-analysis-in-Cell-Ranger->). Graph-based unsupervised clustering was then used to cluster the cells. Loupe browser (https://support.10xgenomics.com/single-cell-gene-expression/software/visualization/latest/what-is-loupe-cell-browser), R packages including cellranger R-kit (<http://cf.10xgenomics.com/supp/cell-exp/cellrangerrkit-PBMC-vignette-knitr-2.0.0.pdf>), complex heatmap^2^, and Geom_violin plots^3^ were used for more in-depth analysis to compare genes expression in each cluster compared to all the other clusters and plot the data.

**Integration of scRNA-seq and proteomic data**

Overlapped and unique genes between proteomic and scRNA-seq data sets for subjects 2003, 2004, and 2005 altogether were identified and represented in Venn diagrams. In addition, Spearman’s correlation analysis between both data sets was conducted and Pearson product-moment correlation coefficient (r) was calculated.

**Integration of gene/protein expression and UPDRS III score**

Next, expression levels of genes/proteins, selected as potential biomarkers based on the functional and pathway enrichment analysis of proteomic data, were assess for their effects on UPDRS III score and the change in scores at 2 and 6 months during treatment normalized to baseline to gauge the severity and progression of PD in patients. To address this question, we conducted Pearson product-moment correlation and multiple linear regression analyses between genes and proteins measured by ddPCR and Western blot, respectively, and UPDRS III score and change from baseline (Statistica, ver13.3, TIBCO Software, Palo Alto, CA).


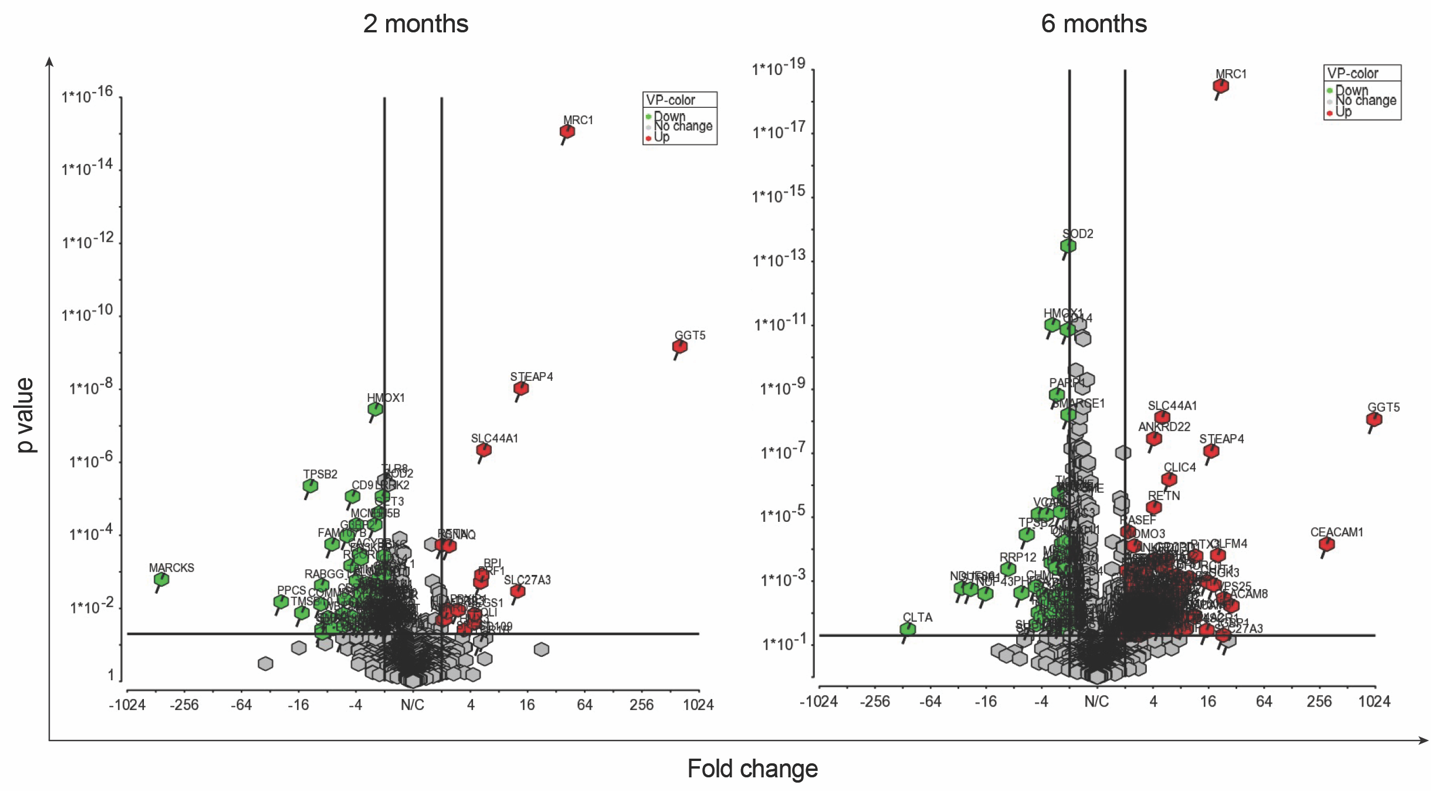
**Fig. S1.** **Differential proteomic analysis of monocytes.** Volcano plots showing the fold change plotted against the p value highlighting significantly changed proteins (red – upregulation and green – downregulation; p ≤ 0.05 and an absolute fold change ≥ 2) in monocytes at 2 and 6 months after the sargramostim initiation compared to baseline before the treatment initiation (n=3 technical replicates). The vertical lines correspond to the absolute fold change of 2, and the horizontal line represents a p value of 0.05.


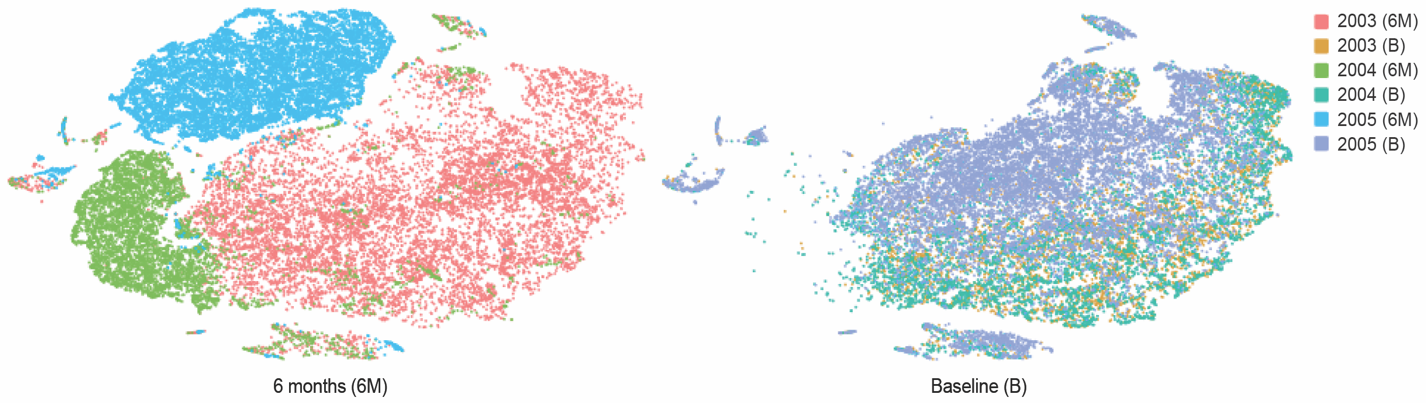


**Fig. S2. Transcriptome signature after sargramostim treatment.** The scRNA-seq data clustering for subjects 2003, 2004, and 2005 after 6 months of sargramostim treatment compared to baseline.

**
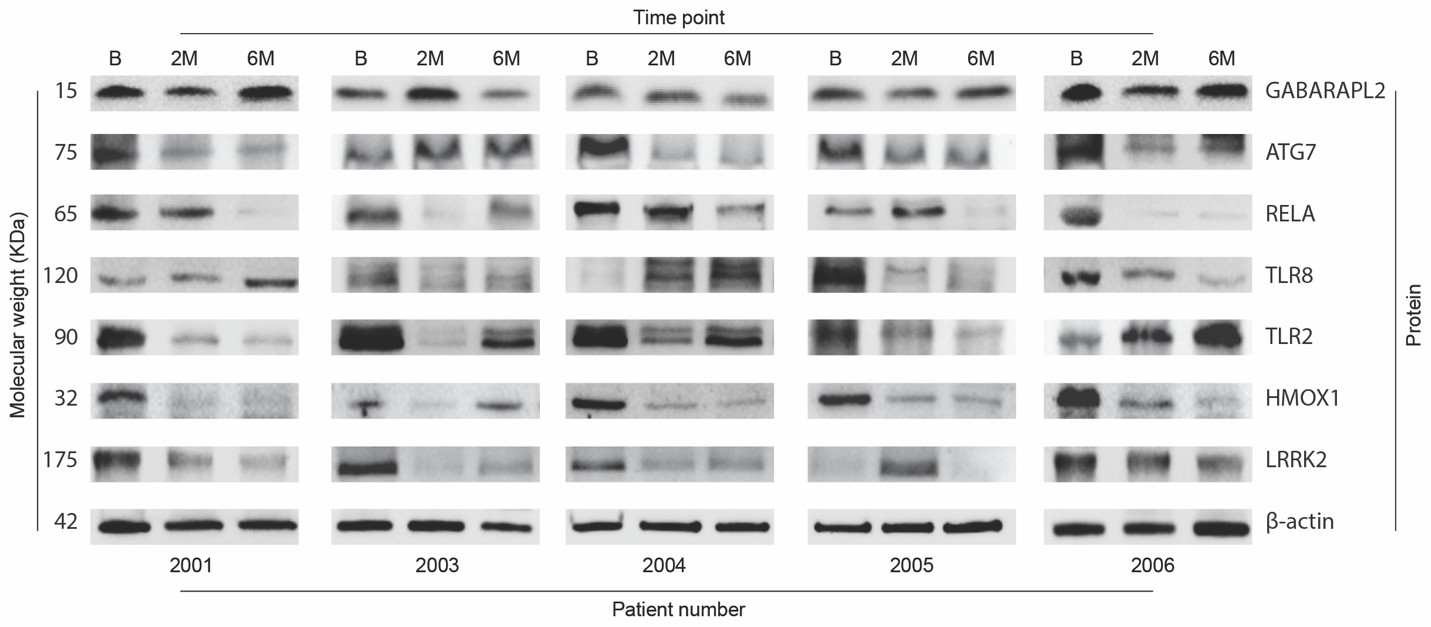
**

**Fig. S3. Western blot analysis of potential biomarkers in monocytes at 2 and 6 months after sargramostim treatment.** Western blot analysis was performed to determine the protein expression of β-actin, LRRK2, HMOX1, TLR2, TLR8, RELA, ATG7, and GABARAPL2 at 2 and 6 months after starting the sargramostim treatment compared to baseline. Representative immunoblots are shown. All experiments were done thrice, and one representative image is shown. B: baseline, 2M: 2 months, and 6M: 6 months.


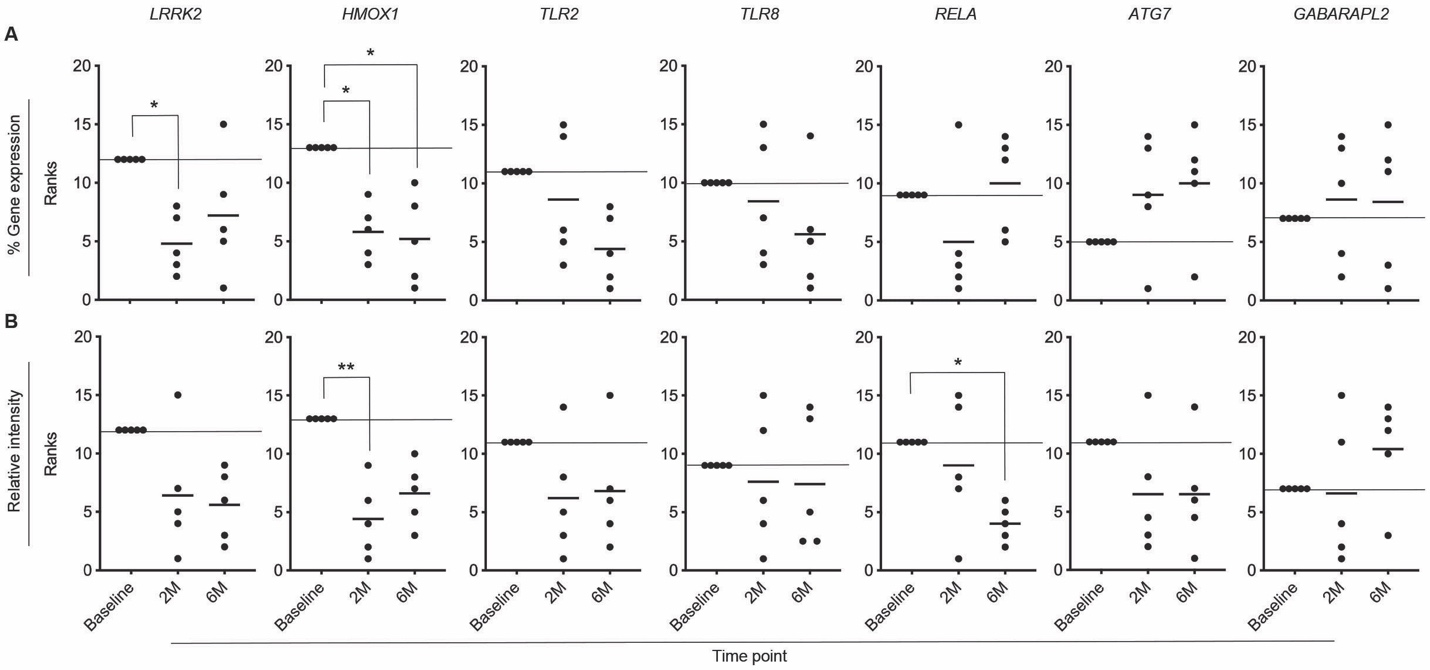


**Fig. S4. Non-parametric analysis of gene and protein expression of potential biomarkers in monocytes at 2 and 6 months after sargramostim treatment.** (A) The ddPCR assay was performed to determine the gene expression of *LRRK2, HMOX1, TLR2, TLR8, RELA, ATG7,* and *GABARAPL2* at baseline (before), and 2 and 6 months after starting the sargramostim treatment. Gene expression was normalized to *HPRT1* and the ddPCR assay was performed 4 times (n=4 technical replicates/subject). (B) Western blot analysis was performed to determine the protein expression of β-actin, LRRK2, HMOX1, TLR2, TLR8, RELA, ATG7, and GABARAPL2 at baseline (before), and 2 and 6 months after starting the sargramostim treatment. Protein expression was normalized to β-actin and densitometric quantification is shown. Western blot analysis was done thrice (n=3 technical replicates/subject). Data are represented as individual values of the mean for each subject’s readings and short horizontal lines in each image represent the mean value for 5 subjects’ readings. Long horizontal line in each image represents baseline expression; values above the line indicate upregulation while values below the line indicate downregulation. Statistical significance between the groups was determined by Kruskal-Wallis test with Dunn’s multiple comparison tests and p ≤ 0.05 was considered significant. ^*^ ≤ 0.05 and ^**^ ≤ 0.005.

**
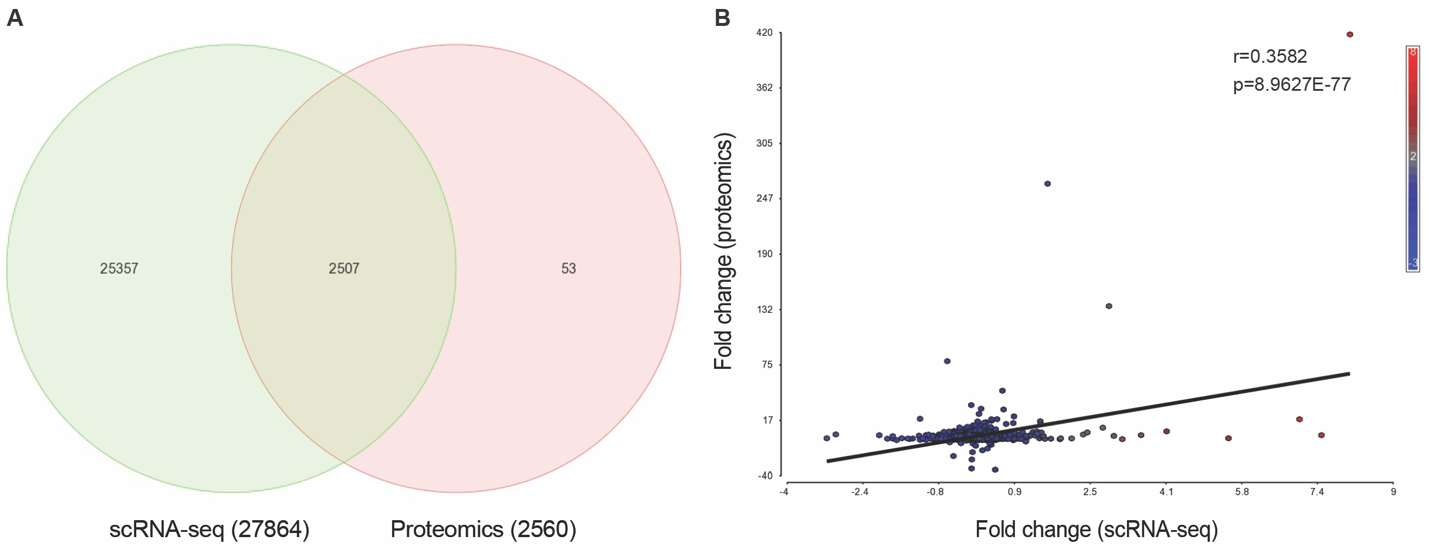
**

**Fig. S5. Integration of scRNA-seq and proteomic data.** (A) Overlapping genes between scRNA-seq and proteomic data sets for patients 2003, 2004, and 2005 at 6 months after sargramostim treatment compared to baseline. (B) Correlation of overlapped genes in both scRNA-seq and proteomic data sets for patients 2003, 2004, and 2005 at 6 months after sargramostim treatment compared to baseline. Correlation was determined using Pearson product-moment correlation coefficient (r).

**Legends for Additional files**

**Additional file 1:** Raw data for proteomic data sets (provided as a separate spreadsheet).

**Additional file 2:** FDR-corrected significantly differentially expressed proteins at 2 and 6 months after the treatment as well as the overlapped FDR-corrected significantly differentially expressed proteins at both time points (provided as a separate Excel sheet).

**Additional file 3:** Raw data for scRNA-seq data set for subjects 2003, 2004, and 2005 (provided as a separate spreadsheet).

**Additional file 4:** Functional and pathway enrichment analysis of 2 month proteomic data set (provided as a separate spreadsheet).

**Additional file 5:** Ingenuity Pathway Analysis (IPA) of 2 month proteomic data set (provided as a separate spreadsheet).

**Additional file 6:** Functional and pathway enrichment analysis of 6 month proteomic data set (provided as a separate spreadsheet).

**Additional file 7:** Functional and pathway enrichment analysis of scRNA-seq data set (provided as a separate spreadsheet).

**Additional file 8:** IPA of 6 month proteomic data set (provided as a separate spreadsheet).

**Additional file 9:** IPA of 6 month scRNA-seq data set (provided as a separate spreadsheet).

**Additional file 10:** STRING analysis of 2 month proteomic data set (provided as a separate spreadsheet).

**Additional file 11:** STRING analysis of 6 month proteomic data set (provided as a separate spreadsheet).

**Additional file 12:** Raw data for 6 month proteomic data set for subjects 2003, 2004, and 2005 (provided as a separate spreadsheet).

**Additional file 13:** Overlapped genes of 6 month proteomic and scRNA-seq data sets for subjects 2003, 2004, and 2005 (provided as a separate spreadsheet).

**Additional file 14:** Unique genes of 6 month proteomic and scRNA-seq data sets for subjects 2003, 2004, and 2005 (provided as a separate spreadsheet).

**References**

1. Gaurav, R.; Mikuls, T. R.; Thiele, G. M.; Nelson, A. J.; Niu, M.; Guda, C.; Eudy, J. D.; Barry, A. E.; Wyatt, T. A.; Romberger, D. J.; Duryee, M. J.; England, B. R.; Poole, J. A., High-throughput analysis of lung immune cells in a combined murine model of agriculture dust-triggered airway inflammation with rheumatoid arthritis. *PLoS One* **2021,** *16* (2), e0240707.

2. Gu, Z.; Eils, R.; Schlesner, M., Complex heatmaps reveal patterns and correlations in multidimensional genomic data. *Bioinformatics* **2016,** *32* (18), 2847-9.

3. Hintze, J. L.; Nelson, R. D., Violin Plots: A Box Plot-Density Trace Synergism. *The American Statistician* **1998,** *52* (2), 181-184.
